# A pilot randomized controlled trial of distance laughter therapy for mothers’ level of depression, anxiety, and parental stress during the COVID-19 pandemic

**DOI:** 10.1371/journal.pone.0288246

**Published:** 2023-07-14

**Authors:** Yejung Ko, Sihyun Park

**Affiliations:** 1 College of Nursing and Health, Kongju National University, Gongju, South Korea; 2 Department of Nursing, Chung-Ang University, Seoul, South Korea; University of Derby, UNITED KINGDOM

## Abstract

The COVID-19 pandemic has led to substantial lifestyle changes worldwide, contributing to heightened psychological stressors such as depression and anxiety. The demands of parental care have also intensified, increasing the risk of caregiver burnout and potential child maltreatment. This study aimed to evaluate the efficacy and feasibility of implementing distance laughter therapy for mothers caring for young children during the pandemic, with a focus on mitigating depression, anxiety, and parental stress. Utilizing a pilot randomized controlled design, 22 participants were divided into two groups—experimental and control groups—and underwent four virtual sessions over two weeks. The experimental group engaged in distance laughter therapy, a technique designed to stimulate self-induced laughter, while the control group viewed a 50-minute entertainment TV show. Both groups experienced a significant decrease in depression and anxiety; however, only the experimental group experienced a significant reduction in parental stress. Nonetheless, the differences in outcomes between the groups were not statistically significant. Participants who engaged in distance laughter therapy reported positive changes across physical, emotional, social, self-perception, and stress-coping domains in exit interviews. Therefore, laughter therapy has an additional benefit of reducing parental stress, which may be particularly useful for mothers primarily responsible for childcare during the pandemic. Future research should investigate the effects of laughter on broader populations and settings and quantify the actual amount of laughter generated.

## Introduction

The emergence of the novel coronavirus infectious disease (COVID-19) has put the health and life quality of the global populace at risk. The fear of infection, coupled with prolonged isolation because of quarantine restrictions and social distancing mandates led to a range of detrimental outcomes, including financial instability [1], conflicts within families and marriages [2], and a rise in violence and crime [3]. Consequently, individuals experienced adverse psychological conditions such as fear, anxiety, and depression [4, 5].

In these challenging times, the female gender has been identified as more susceptible to mental health issues, as many COVID-19 stressors were related with their reproductive functioning [6]; parental stress is a notable stressor in this context. With the closure of schools and child-care facilities across numerous countries during the pandemic, children have spent most of their time at home under parental care [6]. Furthermore, economic hardships, unemployment, a reduction in traditional child-care support, and parents’ diminished leisure time have escalated parental burden, leading to heightened parental stress [7]. Amidst these family-related changes, women in many countries, such as South Korea, are often expected to make sacrifices for their families, as they typically bear the majority of child-care responsibilities [6, 8].

In South Korea, the burden of child-care caused by school closures and a reduction in child-care services increased significantly during the pandemic; however, while paternal responsibility increased from 3.6% to 7.5%, maternal responsibility increased more substantially, from 29.2% to 36.8% [9]. Heightened parental stress can adversely affect health conditions, relationships with partners, and the welfare of children [7, 10]. Parents under considerable stress are less likely to pay attention to their children, participate in their children’s activities, spend time with them, or show interest in their emotional well-being [11]. If parental stress evolves into parental burnout—a state of chronic, overwhelming stress—it could lead to aggressive and neglectful behaviors towards children, potentially culminating in child abuse and neglect [10]. UNICEF, thus, has provided several parenting tips during the pandemic, including strategies to maintain composure as well as manage stress and anger [12]. However, there is a pressing need for more evidence-based interventions to alleviate stress and burnout among parents, particularly mothers.

However, delivering interventions to alleviate psychological stress during the pandemic proved challenging because of the strict social distancing measures implemented in most countries. Nevertheless, owing to the pressing need for strategies to address mental health emergencies, several web-based psychotherapeutic interventions were developed for vulnerable populations to use while maintaining social distancing. This population included COVID-19 patients [13], university students [14], and healthcare workers [15] to help them manage their distress and circumstances. Laughter-inducing therapies formed part of these efforts, targeting vulnerable populations during the pandemic [16].

Laughter is believed to be more than a mere response to humor; it holds potential benefits for both physiological and psychological functions. These benefits include decreased stress hormones; enhanced immune system function; reduced perceived stress, anxiety, and depression; and improved self-esteem, cognitive functions, and quality of life [17, 18]. Moreover, laughter and humor are thought to influence personal development positively across biological, psychological, social, socioeconomic, environmental, and behavioral aspects [19]. Owing to its vital benefits [17], laughter therapy (LT) has been established as a form of cognitive-behavioral therapy and has been applied to a broad spectrum of populations, including healthy adults, children, the elderly, as well as individuals with various health conditions like cancer [20, 21], those undergoing hemodialysis [22], and postpartum women [23]. There are various types of LT, categorized based on whether they incorporate humor or not. Specifically, LT with humor employs humorous videos and materials, while those without humor often include exercises [18]. LT without humor has evolved into group therapies such as laughter yoga [24] and laughter Qigong therapy [25] through integration with various therapeutic methods.

Traditionally, LT has been conducted through face-to-face interactions. However, the current pandemic has prompted the introduction of non-face-to-face LT, facilitated via online platforms. Given its advantages of being non-pharmacological and more readily acceptable to various populations compared to other psychotherapies [17], it is projected that this distanced mode of implementation could be viable. Moreover, LT, known for its stress-reducing and mood-enhancing effects, could serve as a beneficial intervention for individuals experiencing heightened negative moods and stress during the pandemic [26]. In fact, recent trials of online LT have demonstrated efficacy in reducing adverse psychological conditions such as depression, anxiety, stress, and loneliness [27].

However, during the pandemic, the application of LT has been primarily limited to specific populations, particularly nursing students [27, 28]. Furthermore, in most LT literature, control groups typically received standard care or no intervention at all [16, 27]. Consequently, despite prior studies reporting improved outcomes with LT [18], it remains unclear whether the observed effectiveness among isolated individuals during the pandemic is specifically attributable to LT, or whether LT indeed represents the best therapeutic option for them.

In this study, we developed and implemented a distance laughter therapy (DLT) program targeting mothers with young children during the pandemic, facilitated via Zoom (https://zoom.us). Our primary objective was to examine the impact of DLT on depression, state/trait anxiety, and parental stress levels in mothers. We also sought to validate the feasibility and effectiveness of DLT as a coping mechanism for isolated mothers by contrasting it with common pandemic-era activities, such as watching television [29]. Furthermore, upon completion of the intervention, an exit interview was conducted to gain insights into the program’s feasibility based on participants’ perceived changes and feedback. Along with analyzing the exit interview data, the six hypotheses below were tested in this study:

H1-1: DLT will decrease depression levels in the experimental group post-intervention.
H1-2: Compared to those in the control group, the depression levels in the experimental group will decrease post-intervention.
H2-1: DLT will decrease the anxiety levels in the experimental group post-intervention.
H2-2: Compared to those in the control group, the anxiety levels in the experimental group will decrease post-intervention.
H3-1: DLT will decrease parenting stress levels in the experimental group post-intervention.
H3-2: Compared to those in the control group, the parenting stress levels in the experimental group will decrease post-intervention.

## Method

### Participants

This study was a mixed method, parallel, pilot randomized controlled trial (RCT) aiming to examine the feasibility and effectiveness of DLT among women with young children aged under 6 years. Participants’ inclusion criteria were as follows: women who 1) had at least one child under 6 years; 2) had no acute mental health crisis that needed emergency intervention and had not taken psychotropic medications within the past 3 months; 3) could use Zoom at home; and 4) faced difficulties while caring for their children at home during the pandemic. To recruit the participants, web-flyers advertising this study were uploaded on online platforms, including the researchers’ social network services and boards of regional community networks.

Each group had approximately 10 to 11 participants. This number was not optimized for between-group comparisons, as achieving 80% power (effect size = .5, α = .05) to detect differences between two independent groups requires a minimum sample size of 51 per group. However, because of the logistical challenges in managing larger groups in a Zoom-based intervention and considering the importance of participant interaction within our program, we opted for a smaller sample size. Eventually, 22 participants were recruited. Participants voluntarily provided written informed consent via email; thereafter, the online baseline survey was conducted.

### Intervention: Distance laughter therapy

Table 1 shows the specific contents of the DLT performed in this study. The DLT contents were developed by this study’s first author and a certificated clinical laughter therapist. The first author, who is certificated as a psychiatric nurse specialist, had prior experience in developing and applying LT to immigrant women in South Korea to reduce their acculturation-related stress responses [30]. The clinical laughter therapist, who performed the LT intervention in this study, had multiple experiences of conducting LT to various population groups.

**Table 1:** Contents of the distance laughter therapy.

Stage	Contents		Duration
Introduction	Opening (Self introduction and greetings) Facial stretching and raising mouth corner		10min
Implement	Part 1	Practicing various laughter techniques	30min
	Part 2	Bursting into laughter	
	Part 3	Laughter with clapping	
	Part 4	Laughter with singing and dancing	
Wrap-up	Expressing oneself with laughter “This is me” (1^st^ session) Reinforcing the positivity with laughter “I thank to…” (2^nd^ session) Increasing self-esteem with laughter “I like me because…” (3^rd^ session) Complimenting each other with laughter “Yes, I hear that a lot” (4^th^ session)		5min
Evaluation	Relaxing and sharing the emotion Saying good-bye		5min

The DLT employed in this study had several distinct features. First, we instructed all participants to adjust their Zoom settings to display all attendees, including the therapist, on a single screen. They were also required to keep their cameras on and show their faces throughout the session, enabling them to see their own and others’ faces during the program.

Second, we employed a simulated laughter approach. Literature on laughter therapy frequently differentiates between “simulated” and “spontaneous” laughter [17, 18]. Spontaneous laughter is a natural response to humorous stimuli, such as jokes [18]. In contrast, simulated laughter refers to intentional or voluntary laughter, which can be enacted without any humorous stimuli [18]. Both types of laughter have been employed in intervention studies; however, recent meta-analytic research indicates that simulated laughter may be more effective [18].

Finally, in our study, the DLT specifically targeted positive, humor-free laughter, in line with the Humour-Laughter-Affect (HuLA) model [19]. We designed the program to encourage self-induced laughter in a mutually supportive environment, facilitated by interactions among participants.

The DLT in this study comprised four sessions; it was performed twice per week for two weeks. It was based on previous meta-analysis [31] reporting that four-session programs showed higher effect size compared to three-session programs, and the number of sessions per week was considered more important in enhancing the program outcome than was the duration of the sessions. Each DLT session comprised four stages—introduction, implementation, wrap-up, and evaluation. The program comprised various simulated laughter techniques followed by facial stretching, such as laughter with clapping, singing, and dancing. Each session was about 50 min-long.

### Conditions for control group

The control group was asked to watch a popular South Korean 50-minute entertainment TV show titled “Infinite Challenge” via their personal computers, while the experimental group participated in four sessions of group-based laughter therapy. The treatment provided to the control group was the same in length and frequency as the one provided to the experimental group; further, the treatment was delivered in the same condition: at home.

### Study procedure

One research assistant (RA), who was not involved in this study, randomly divided the 22 participants into two groups: an experimental group (n = 11) and a control group (n = 11), using a research randomizer site (http://www.randomizer.org). We applied the double-blind method; therefore, the therapist who performed the LT and the participants were blinded to allocation. Two researchers of this study were also blinded to allocation, as they were not involved in the programs until all sessions were completed and post-test performed.

Before the intervention, the RA sent a URL to participants as well as the participation schedule (date and time). Using the URL sent, the experimental group accessed a Zoom classroom for LT and the control group accessed the TV show. After completing two-weeks of intervention, a post-test of the two groups was conducted online. Among the 22 participants, five were excluded: one participant had registered for this study but did not show up for the baseline survey, and four participants in the experimental group had withdrawn because of their busy schedules. No participant from the control group was excluded.

**Fig 1:**
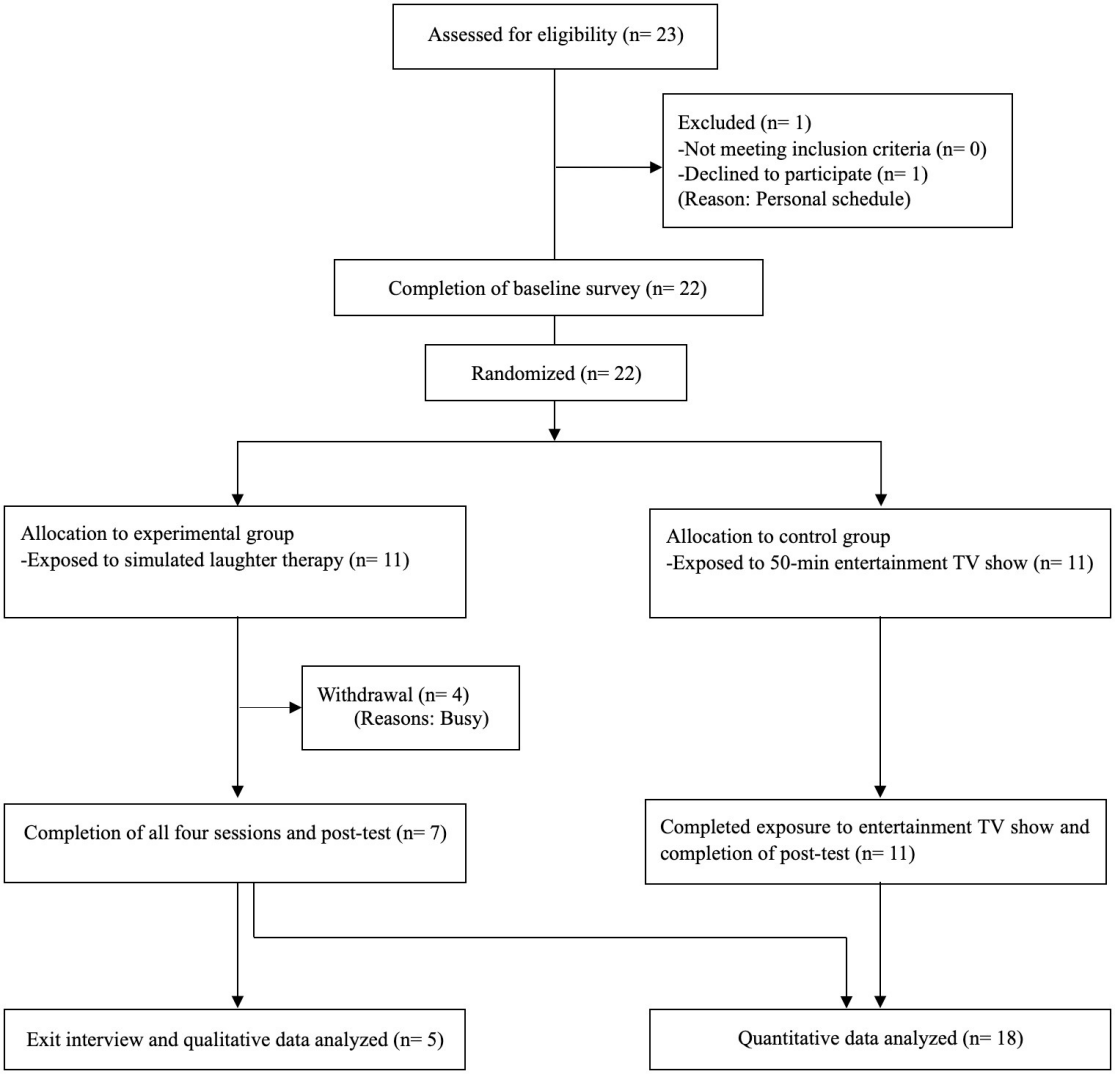
Flow diagram of study procedure.

After the intervention, the researchers asked participants in the experimental group to participate in an exit phone interview, and five participants volunteered. Using a semi-structured questionnaire, four questions were asked: 1) what was your participation experience in the four DLT sessions conducted through a virtual meeting?; 2) what did you feel about DLT, whether good or bad?; 3) what do you think about the program’s effects, if any, and why?; 4) do you perceive any changes after participating in this program, compared to before? Each interview lasted between 30 and 40 minutes. All interviews were audio-recorded and had verbatim transcription. The participants who completed the study were compensated with 17.33 USD (20,000 KRW), and those who participated in the exit interview were offered an additional 8.67 USD (10,000 KRW). The specific procedure is illustrated in Fig 1.

### Measurement

#### Depression

To assess the depression, the Korean version of the CES-D (Center of Epidemiologic Studies Depression Scale) was used [32]. It comprised 21 items, with a 4-point Likert scale rating from 1 (no symptom presence) to 4 (most of all the time). Higher scores indicated higher levels of depression. The Cronbach’s alpha (α) of this scale in this study was .93.

#### State/Trait anxiety

State and trait anxiety was measured based on the Korean-version of the State-Trait Anxiety Inventory [33]. It comprised 40 items in total: 20 items measured state anxiety and the other 20 measured trait anxiety. It was rated on a 4-point Likert scale from 1 (almost never) to 4 (almost always). Higher scores indicated higher levels of anxiety. The Cronbach’s α of state and trait anxiety scales were .96 and .94, respectively.

#### Parental stress

Parental stress was assessed using Korean Parenting Stress Scale, a Korean cultural-specific tool developed by Kim and Kang [34]; however, we revised the scale to fit the COVID-19 pandemic situation before applying it to the participants in this study. The scale comprises 32 items, and each item was scored on a 5-point Likert scale from 1 (not at all) to 5 (severely). Item’s examples included “It’s physically too hard to take care of a child,” “I’m not sure if I can raise my child well,” and “There are times when I want to run away from a child.” The Cronbach’s α of this tool was .95.

### Data analysis

Quantitative data were analyzed using SPSS version 26. A Shapiro-Wilk test, which is recognized for its power in detecting non-normality in continuous data with a small sample size (n < 50), was performed [35]. The results confirmed that the data were normally distributed. Descriptive statistics, including frequencies, proportions (%), means, and standard deviations (SD), were calculated to examine the demographic characteristics. Chi-square tests and independent t-tests were utilized to evaluate the homogeneity of demographics and baseline outcomes between the experimental and control groups. To determine changes pre- and post-DLT program within each group, paired t-tests were performed. Independent t-tests were conducted for between-group comparisons. The dataset did not contain any missing values.

Qualitative data were analyzed using a conventional content analysis [36]. Interview transcriptions from five interviewees were read twice by the researchers, and their opinions and experiences regarding the DLT program were highlighted. The highlighted parts were then open coded using a line-by-line coding technique, and 228 codes were found. Similar codes were categorized as the broader concepts, and similar categories were merged into themes. In total, 2 themes, 7 categories, and 14 sub-categories emerged.

### Ethical consideration

The current research was conducted and reported by following the CONSORT 2010 [37], and it was approved by the institutional research board of C University before recruiting the participants (IRB No.1041078-202103-HRSB-064-01). Written informed consent was obtained from the participants via email. As this study was conducted during the COVID-19 pandemic outbreak, we followed the Ethical Standards for Research during Public Health Emergencies developed by the World Health Organization [38].

## Results

### Demographic characteristics of participants

The mean age of the participants was 35.41 years old (SD: 4.67), and the average number of children was 1.64 (SD: .58). All participants were married, and the majority of them identified as non-religious (72.7%). Moreover, 54.5% were housewives and 31.8% reported that they were taking time off from their work at the time of the survey. Two participants had part-time jobs and one had a full-time job. Their perceived socioeconomic status (SES) varied from high to middle-low: more than half of the participants (59.1%) identified their SES as a middle, and none of them had low SES. On average, they spent 13.16 hours per day on childcare (SD: 5.19).

### Homogeneity between experimental and control groups

Table 2 shows the test results of the homogeneity between the experimental and control groups. There were no significant differences between the two groups in terms of their demographic characteristics as well as the baseline outcomes. Therefore, we assumed that the experimental and control groups were homogeneous.

**Table 2:** Homogeneity test of demographic characteristics and baseline outcomes between the experimental and control groups (n = 22).

Characteristics & Baseline Outcomes	Total (n = 22)		Experimental Group (n = 11)		Control Group (n = 11)		P [95% CI]
	n (%) or M±SD		n (%) or M±SD		n (%) or M±SD		
Age	35.41	± 4.67	34.18	± 5.46	36.64	± 3.59	.227 [-6.56, 1.65]
Number of children	1.64	± .58	1.45	± .52	1.82	± .60	.146 [-.87, .14]
Marital status							-
Unmarried	0	(0)	0	(0)	0	(0)	
Married	22	(100)	11	(100)	11	(100)	
Divorced	0	(0)	0	(0)	0	(0)	
Having religion†							.635
Yes	6	(27.3)	2	(18.2)	4	(36.4)	
No	16	(72.7)	9	(81.8)	7	(63.6)	
Employment status							.370
Full-time	1	(4.5)	1	(9.1)	0	(0)	
Part-time	2	(9.1)	0	(0)	2	(18.2)	
Taking time off	7	(31.8)	4	(36.4)	3	(27.3)	
Housewife	12	(54.5)	6	(54.5)	6	(54.5)	
Socioeconomic status							.279
High	1	(4.5)	1	(9.1)	0	(0)	
High-middle	2	(9.1)	2	(18.2)	0	(0)	
Middle	13	(59.1)	6	(54.5)	7	(63.6)	
Middle-low	4	(18.2)	2	(18.2)	2	(18.2)	
Low	2	(9.1)	0	(0)	2	(18.2)	
Marital satisfaction	3.61	± .98	3.59	± 1.07	3.63	± .94	.917 [-.94, .85]
Paternal involvement	3.64	± 1.15	3.70	± 1.37	3.57	± .96	.789 [-.91, 1.19]
Average time for childcare/day (hrs)	13.16	± 5.19	13.00	± 6.18	13.32	± 4.28	.890 [-5.05, 4.41]
Depression	2.16	± .51	2.21	± .62	2.10	± .40	.613 [-.35, .57]
State anxiety	2.42	± .61	2.47	± .64	2.37	± .61	.699 [-.45, .66]
Trait anxiety	2.38	± .61	2.49	± .64	2.27	± .60	.417 [-.33, .77]
Parenting stress	3.30	± .80	3.28	± .91	3.31	± .73	.915 [-77, .70]

^†^p values were calculated through two-tailed Fisher’s exact test

### Impacts of distance laughter therapy

Table 3 shows the changes in depression, state/trait anxiety, and parenting stress before and after applying the intervention. Within the experimental group, all outcome-mean scores of depression (p = .038), state/trait anxiety (p = .002/p = .025), and parenting stress (p = .034) were significantly decreased. However, the control group also showed significant decrease in depression levels (p = .028) and state/trait anxiety (p = .006/p = .003) but not in parenting stress (p = .064). The differences, before and after the intervention, in all outcome variables in the experimental group were greater compared to those in the control group; however, none was statistically significant (p = .193, p = 116, p = .158, and p = .061, respectively).

**Table 3:** Comparison of the level of depression, state/trait anxiety, and parental stress before and after applying the distance laughter therapy (n = 18).

Outcome variables	Experimental group (n = 7)				Control group (n = 11)				t‡	p‡ [95% CI]
	Pre (M±SD)	Post (M±SD)	Δ (Post-Pre)	p† [95% CI]	Pre (M±SD)	Post (M±SD)	Δ (Post-Pre)	p† [95% CI]		
Depression	2.19 (± .62)	1.65 (± .44)	-.54 (±.54)	.038 [.04, 1.05]	2.10 (± .40)	1.76 (± .50)	-0.34 (±.44)	.028 [.04, .63]	-.89	.386 [-.70, .28]
State anxiety	2.51 (± .76)	1.79 (± .62)	-.72 (±.37)	.002 [.38, 1.06]	2.37 (± .61)	1.90 (± .64)	-0.47 (±.45)	.006 [.17, .77]	-1.24	.233 [-.69, .18]
Trait anxiety	2.54 (± .70)	1.88 (± .59)	-.66 (±.58)	.025 [.12, 1.20]	2.27 (± .60)	1.84 (± .67)	-0.43 (±.36)	.003 [.18, .67]	-1.04	.316 [-.70, .24]
Parenting stress	3.52 (± .93)	2.64 (± 1.14)	-.88 (±.85)	.034 [.09, 1.67]	3.31 (± .73)	2.97 (± 1.06)	-0.34 (±.55)	.064 [-.02, .71]	-1.63	.122 [-1.23, .16]

^†^within group comparison;

^‡^between groups comparison

### Findings from the exit interview data

The themes and categories from the exit interview are listed in Table 4. Two major themes from the exit interview were perceived changes after participating in the DLT and program feedbacks.

**Table 4:** Themes and categories from the exit interview data.

Themes	Categories	Sub-Categories
Perceived changes	1. Physical changes	1-a. Feeling relaxed and energized
		1-b. Transition from awkward to natural smile
	2. Emotional changes	2-a. Simulated laughter evolving into genuine happiness
		2-b. Alleviation of depressive emotions through laughter
	3. Social changes	3-a. Improved social relations
	4. Changes in self-perception	4-a. Rediscovering lost smiles
		4-b. Boosting self-esteem through self-focus
	5. Changes in stress coping	5-a. Coping with stress through laughter
		5-b. Committing to change and further efforts
Program feedbacks	1. Strengths and limitations of web-based distance intervention	1-a. Ease and convenience of participation
		1-b. Reduced sense of intimacy
	2. Seizing the opportunity for social interaction amid the pandemic	2-a. Connecting with fellow mothers sharing similar experiences
		2-b. Fostering rapport with other participants
		2-c. Experiencing joy in social interaction during the pandemic

#### Perceived changes

After participating in DLT, participants reported numerous changes, encompassing physical, emotional, social, self-perception, and stress-coping domains. Under the physical changes category, two sub-categories, “feeling relaxed and energized” and “transition from awkward to natural smile,” emerged. Participants reported that the experiences from the program fostered a sense of bodily comfort and energy, and they observed muscle relaxation. Participant R2 described her experience, stating, *“I was trained to laugh with my whole body*, *not just a simple ‘hahaha*.*’ I feel as though my entire body has awakened*, *filled with positive energy*.*”*

Regarding emotional changes, two sub-categories, “simulated laughter evolving into genuine happiness” and “alleviation of depressive emotions through laughter,” emerged. Participants initially believed that laughter was solely a result of feeling happy, but they discovered that the act of laughing itself lifted their moods. Participant R3 shared, *“I forced myself to laugh*, *and surprisingly*, *it led to genuine enjoyment*.*”* The contagious nature of laughter also played a role in enhancing feelings of joy and happiness. Participant R1 stated, *“After the first class*, *I think I became really happy*. *I met a friend after the session*, *and they even said I looked like I was in a good mood*.*”*

Regarding the social aspect, a single sub-category, “improved social relations,” emerged. Participants reported enhancements in their relationships, particularly with family members, after participating in DLT. For instance, participant R3 shared a joyful experience with her family using what she learned from the therapy: *“I showed my kid the laughter exercises I learned*. *They really got a kick out of copying them*. *I even taught my husband*, *and we had a good laugh together*.*”* Participant R4 also shared the positive feedback from her family regarding her transformation, stating, *“I think my kids are warming up to the new me*. *My husband even asked them*, *‘Did something good happen to mom*?*’”*

Numerous participants also reported shifts in their self-perception, falling into two sub-categories: “rediscovering lost smiles” and “boosting self-esteem through self-focus.” As they had primarily identified as mothers and wives for an extended period, the program enabled them to reconnect with their individual identities. Observing their smiles on screen, participants realized they had rediscovered their lost smiles. Participant R4 reflected, “*I remembered that I was a cheerful person when I was younger*. *It’s time to gradually change*, *to cheer up again*.*”*

Lastly, participants discussed their improved stress-coping abilities, encapsulated by two sub-categories: “managing stress through laughter” and “commitment to change and continued efforts.” When feeling overwhelmed, they applied the laughing techniques learned from the DLT, finding it effective in mitigating their stress. Participant R5 noted, *“The program was quite helpful*. *Whenever I felt agitated*, *I used the techniques I learned*, *and it helped me cool down*.*”* All participants expressed a strong desire to participate in DLT again if given the opportunity. Participant R3 was particularly keen on further DLT classes, stating, *“I’ve written down the various laughter techniques I learned in this program and use them regularly*. *I’ve even bought and started reading books on laughter therapy*.*”*

#### Program feedbacks

Participants provided feedback on their experiences with DLT. One key aspect they discussed involved the advantages and drawbacks of the web-based distance approach. All participants expressed high satisfaction with this method because of its convenience and comfort—they appreciated not needing to dress up, arrange childcare, or commute. They particularly noted its appropriateness during the pandemic, when social gatherings were restricted. Participant R2 commented, *“It was convenient because I didn’t have to go anywhere or meet anyone*. *It was also quite easy to participate*.*”* The participants also found value in seeing their own smiling faces directly on the Zoom screen, an opportunity not typically available in offline programs. However, two participants expressed that an in-person LT might have been more enjoyable and could have fostered a closer connection with other participants.

The second feedback theme involved gratitude for the opportunity to interact with other mothers with young children. Given their shared experiences and challenges while caring for young children during the COVID-19 pandemic within Korean society, participants noted that they could easily establish rapport with like-minded individuals through shared understanding and empathy. Participant R3 remarked, *“I believe there was a ‘motherly’ bond among the participants*.*”*

## Discussion

We developed and applied DLT using a virtual meeting technology for mothers caring for their young children during the pandemic. We evaluated its impact on reducing levels of depression, anxiety, and parental stress and assessed its application feasibility. We found that parental stress levels during the pandemic were notably more severe compared to those in previous years. The mean score for mothers’ parental stress in this study was 3.30 and were higher than the scores reported by Korean mothers with young children in 2002 and 2007, which were 2.51 and 2.73 respectively, using the same scale [39]. This indicates that the target population in this study was in significant need of care and intervention.

In this study, participants in the experimental group demonstrated a significant decrease in their depression and anxiety levels after completing the DLT. These findings align with Van der Wal and Kok [18], who highlighted that reductions in depression, stress, and anxiety levels were the most common outcomes in LT studies, hence affirming hypotheses H1-1 and H2-1. Our results are consistent with those of two previous RCTs that evaluated the impact of online-based LT during the pandemic. One study reported a significant decrease in depression levels but a non-significant decrease in anxiety and stress levels after eight sessions of laughter therapy with nursing students, each session lasting 40–45 minutes [27]. The authors suggested that the anxiety and stress levels had plateaued and become chronic because of the protracted duration of the pandemic, a finding that contrasts with our results and those of another previous study that reported significant reductions in anxiety following ten sessions of online LT [28].

However, the degree of decrease in depression and anxiety levels did not differ significantly from that observed in the control group, who were exposed to an entertainment TV show. Therefore, hypotheses H1-2 and H2-2 were not supported. This might be attributed to the positive effects that the general TV entertainment show had on the participants’ depression and anxiety levels.

Parental stress was significantly reduced in the experimental group post-intervention, contrasting with the control group where it remained relatively unchanged. As such, Hypothesis H3-1 is supported. This suggests that, unlike solitary TV viewing, simulated laughter and group interaction may help alleviate parenting-induced stress. However, since the degree of between-group difference did not reach statistical significance, our interpretation requires caution, and thus Hypothesis H3-2 is not supported.

Considering our quantitative results, we cannot definitively claim that DLT is the superior choice for mothers, particularly given the effort and resources required for participation compared to simply watching TV. Indeed, a substantial number of participants (n = 5) withdrew from the experimental group because they were “busy,” while none withdrew from the control group. Nevertheless, owing to our small sample size, the quantitative findings should be interpreted with caution and require confirmation through future studies. Additionally, as we did not measure or compare the amount of laughter between the experimental and control groups, we could not ascertain the frequency of laughter in the control group or how effective the amount of laughter was in reducing depression, anxiety, and parental stress.

Despite the limited effects of DLT, as indicated by the quantitative data, the study participants reported positive experiences and changes from multiple perspectives, including physical transformations. Specifically, they mentioned muscle relaxation and feeling energized, which aligns with Yim’s findings [17] regarding the outcomes of laughter such as muscle relaxation and improved circulation. This is also consistent with other physical effects reported in prior studies, such as increased heart rate, decreased parasympathetic activity [40], relaxation [24], and reduced fatigue and pain [18, 21]. Additionally, participants reported an uplifted mood, which contributed to alleviating their negative psychological conditions. They described how, even though the laughter was initially fake and forced, it evolved into genuine feelings of happiness and pleasure, effectively reducing their sense of depression and stress.

A noteworthy observation was the effectiveness of simulated laughter as a self-care strategy, given its ease of self-implementation and repetition. In contrast, spontaneous laughter, which is not as easily self-induced or repetitively invoked, may not be an effective self-care strategy. This contrast might explain why simulated laughter has consistently shown more positive outcomes compared to spontaneous laughter in previous meta-analyses [18].

This study’s participants reported an increase in feelings of happiness and joy, which they attributed to observing their own and others’ laughter-filled faces. Furthermore, participants noted positive changes in their children and spouses who observed their bouts of laughter and merriment. This phenomenon may reflect the “contagion effect of laughter” noted in previous studies, which assert that witnessing or hearing another person’s laughter can trigger one’s laughter [24, 41]. Gonot-Schoupinsky and Garip also found that laughter was contagious or self-contagious among many participants in their Laughie laughter prescription program, as evidenced by one participant who laughed upon hearing their own laughter [42]. In this context, Zoom emerged as an effective tool for LT as it enabled participants to observe their own and others’ facial expressions during the DLT sessions.

The ongoing pandemic has significantly curtailed social interaction, leaving health professionals struggling to adapt existing interventions for isolated individuals. Even though the DLT program in this study did not outperform entertainment TV shows in terms of impact, we still advocate for its use as an intervention for isolated mothers. Using virtual meeting technology, the DLT has showed a significant effect in alleviating levels of depression, anxiety, and parental stress among mothers, and it has garnered positive feedback. Participants voiced high satisfaction levels, appreciating the opportunity to participate comfortably in an enjoyable environment. They also expressed appreciation for the chance to meet and interact with like-minded people through the DLT during the pandemic. Moreover, according to the Balance between Risks and Resources theory, parental burnout can occur when the demands of parenting surpass available resources [43]. In this regard, DLT can serve as a valuable tool for parents dealing with heightened parenting demands and responsibilities during the COVID-19 era. Beyond the pandemic, DLT could also be beneficial for those with limited resources and those isolated at home with children.

However, it is important to note that the findings of this study may not be readily applicable to diverse populations with varying ethnic backgrounds in different geographic locations because of the limited sample of women from a single ethnicity. Moreover, the small sample size utilized in this study necessitates cautious interpretation of the results obtained from quantitative data analysis. It is also worth mentioning that the effectiveness of the program on unmarried or divorced women remains unexplored, as our study did not include this demographic. Additionally, as we did not conduct a quantitative follow-up to assess the duration of the program’s effects beyond completion, we cannot determine how long these effects persist. Another factor to consider is that the humorous content of the TV show exposed to the control group may have had an impact, as it is known to have its own benefits, including solitary laughter [44]. This potential confounding variable could have been addressed if we had measured and compared the amount of laughter during the DLT program between the experimental and control groups; however, this was not possible in our study.

Therefore, it is imperative that further research is conducted to address these limitations. Specifically, future studies should aim to test the efficacy of DLT using a larger sample size to ensure sufficient statistical power. Additionally, it is crucial to explore the effects of DLT while considering participant characteristics such as parents’ gender, age, relationship with their children, and existing social support systems. Finally, it is necessary to investigate the impact of DLT on diverse populations residing in various geographical regions, as well as its long-term effects.

## Supporting information

S1 Checklist CONSORT 2010 checklist of information to include when reporting a randomised trial*. (DOC)

S1 File (PDF)

S2 File (PDF)
