# Supplementary material for: A pilot randomized controlled trial of distance laughter therapy for mothers’ level of depression, anxiety, and parental stress during the COVID-19 pandemic
Source: PLoS One. 2023 Jul 14;18(7):e0288246. doi: 10.1371/journal.pone.0288246 (PMC10348554; doi:10.1371/journal.pone.0288246)
Supplement: S1 File — (PDF) [file pone.0288246.s002.pdf]

연 구 계 획 서

▶ 연구 과제명 :

COVID-19 시기 영유아기 자녀 어머니의 불안, 우울 및 양육스트레스 감소를 위한 비대면 웃음치료 프로그램의 적용 및 효과 검증

1. 연구 배경

2020년 초 경 발생된 코로나바이러스감염증-19 (이하 COVID-19)로 인하여 세계보건기구(WHO)가 감염증 최고 경보단계인 팬데믹을 선언하면서 여러 국가들이 감염예방 및 COVID-19의 영향을 최소화하고자 고군분투하고 있다. 이에, 여러 국가와 도시들은 바이러스의 감염전파 속도를 늦추고자 사람의 이동과 접촉을 제한하기 위해 사회적 거리두기 정책부터 도시 봉쇄(Shut down)까지 다양한 정책들을 펼쳤다. 한국의 경우도 마찬가지로 정부가 주도하는 사회적 거리두기 정책을 펼치고 있으며 이로 인해 국민들은 '언택트(untact)'라는 새로운 시대를 경험하고 있다. 즉, 교육, 모임, 소비, 근무 등 대부분의 일상이 비대면 및 원격방식으로 전환되고 있으며 그로 인해 사람들 간의 대면 모임과 교류는 점차 줄어들고 있고 고립을 경험하는 인구집단이 늘어나고 있다.

이러한 시대적인 추세로 인해 최근 보건의 이슈는 감염병 예방, 역학 및 백신에 초점이 맞춰져왔으며 상대적으로 정신건강에 대한 부분이 간과되었던 것이 사실이다. 그러나 최근 COVID-19 유행 초반 여러 정신건강 학자들이 경고한 바 대로 언택트로 인한 불안, 우울, 스트레스, 불면증, 분노, 공포 등 다양한 대중의 정신건강문제들이 수면 위로 떠오르고 있고 이는 '코로나 블루'라는 새로운 용어까지 만들어내게 되었다(Galea, Merchant, & Lurie, 2020).

실제로 2003년 SARS-CoV나 2015년 MERS 유행 시에도 의료기관에서 일하는 의료진들을 중심으로 PTSD나 우울, 불안과 같은 정신과적 증상이 보고되었다(Torales et al., 2020). 또한 확진자를 접촉한 사람이나 감염자에 대한 대중들의 낙인과 회피 및 공포적 시선으로 인해 당사자들은 더욱 고통을 겪었다고 알려져 있다(Torales et al., 2020). COVID-19의 경우 앞선 두 바이러스 유행사례에 비해 월등히 높은 감염사례가 발생하고 있고 유행기간이 상당히 긴 점을 감안할 때 향후 정신건강위기의 대규모 발생이 예측되는 바이다. 이에, COVID-19와 관련한 정신건강위기 예방 및 회복을 위한 비대면 방식의 중재가 시급하다고 하겠다.

특히 영유아를 양육하는 여성의 경우 정신건강의 위기가 높을 것으로 예상한다. 본 연구자의 직전 연구에서 영유아를 양육하는 여성들은 COVID-19시대에 극심한 정신적 스트레스를 겪음을 보고하였다. 특히 이는 COVID-19가 가정의 경제적 문제, 육아도움기관이나 주변 도움인의 부재, 사회적 불안의 증가에 영향을 주고 이것이 여성들의 장시간의 양육, 주변으로부터의 전통적 아내/엄마 역할에 대한 기대 증가, 가정 내에서의 고립, 파트너와의 갈등 및 폭력, 사회적 지지의 감소 등으로 이어져 여성들의 정신건강 및 삶의 질에 부정적 영향을 초래하였다.

어머니의 높은 스트레스로 인해 발생되는 문제들은 더 나아가 자녀와 가정 그리고 사회로 번져갈 수 있기 때문에 사회적 관심과 중재가 필요하다. 몇 년 전부터 양육스트레스의 감소를 위한 중재 방안으로 교육청에서 부모교육, 학부모 연수와 같은 교육 중심 서비스의 지원을 강화하고 있다. 또한 여성가족부에서 생애주기에 따라 단계별 가족교육을 활성화 하여 여성의 사회생활과 양육의 부담을 덜어주는 환경을 마련하고자 노력하

고 있다. 그러나 부모교육과 양육기술과 관련된 교육의 제공을 통해서 양육부담을 줄이는 것은 한계가 있다는 연구결과를 통해 어머니의 양육 스트레스 감소를 위한 심리지원의 중요성이 강조되고 있다(Jung & Lee, 2018). 이러한 관점에서 미술치료(Jung & Lee, 2018), 음악요법(Won & Kang, 2018), 원예요법(Yeu, Bae, Woo, Kim & Kim, 2020) 등이 어머니의 양육스트레스와 관련된 심리중재로 연구되었으며 유의한 효과가 있는 것으로 나타나 다양한 심리 중재에 대한 연구의 확대가 필요하다.

웃음치료란 심리행동치료 중 하나로써 웃음을 유발하는 다양한 기제를 사용하여 대상자의 웃음을 이끌어내고 이를 통해 긍정적인 감정을 도출해내는 중재이다. 웃음은 자발적 웃음(spontaneous laughter)과 시뮬레이션 된 웃음(simulated laughter)으로 구분한다(Mora-Ripoll, 2011). 자발적 웃음은 농담과 같은 유머러스한 자극에 의해 유발되는 웃음으로 일반적으로 안와 주변의 수축을 유발시키며 미소 또는 유머 웃음(humorus laughter)로 불리며 진짜 웃음으로 인식된다. 시뮬레이션 된 유머는 자신에 의해 촉발되며 유발하고 의식적으로 촉발되기 때문에 가짜 웃음(fake laugh)으로 불린다(Foley, Matheis, & Schaefer 2002). Wal & Kok(2019)의 연구에서 자발적인 웃음보다 시뮬레이션 된 웃음을 활용한 중재가 더 효과적인 것으로 나타났다. 웃음치료는 비자발적 웃음(Simlated laughter therapy)을 적용한 프로그램의 형태로 다양한 대상자인 산후우울여성(Ryu, Shin, &Yang, 2015), 암환자(Morishima et al.,2019), 노인(Kuru Alıcı, ZorbaBahceli, & Emiroğlu, 2018) 등에 적용되었으며 근긴장을 완화시키고, 스트레스 호르몬을 감소시키며, 정신심리증상을 완화시키고 자아존중감 및 삶을 질을 증진시키는 등 여러 신체, 정신, 사회적 건강결과들에 긍정적인 영향을 주는 것으로 나타났다(Yim, 2016). 웃음치료는 특히 특별한 도구나 장소가 필요하지 않기 때문에 높은 적용성이 있는 것으로 알려져 있다. 그러나 웃음치료를 비대면으로 시도한 연구는 부재하며, 현 시대와 같이 언택트를 기반으로 한 정신건강중재가 절실한 요즘 이와 같은 연구의 필요성이 더욱 대두되고 있다고 할 수 있겠다. 이에, 본 연구에서는 비대면 방식의 웃음치료 프로그램을 COVID-19로 인해 장시간 양육을 하는 여성들에 적용하여 그들의 불안, 우울 및 양육스트레스 감소에 미치는 효과를 검증하고자 한다.

2. 연구 목적

본 연구의 목적은 COVID-19로 인한 사회적 거리두기 시기에 비대면 웃음치료의 적용이 영유아자녀를 둔 어머니의 불안, 우울 및 양육스트레스에 미치는 영향을 검증하는 것이다. 본 연구를 통해 검증할 가설은 다음과 같다.

1) 비대면 웃음치료를 적용한 실험군은 대조군에 비해 불안이 감소할 것이다.
2) 비대면 웃음치료를 적용한 실험군은 대조군에 비해 우울이 감소할 것이다.
3) 비대면 웃음치료를 적용한 실험군은 대조군에 비해 양육스트레스 정도가 감소할 것이다.

또한 본 프로그램 참여의 경험을 Exit interview를 통해 파악하고자 한다.

3. 연구 실시 기관명 및 주소

연구 실시기관: 중앙대학교

연구 실시기관 주소: 서울시 동작구 흑석로 84 중앙대학교 간호학과

4. 연구 지원 기관

본 연구는 국립공주대학교 교내연구비로 수행할 예정이다.

5. 연구 책임자, 공동연구자, 담당자 정보

	이름	소속	직위	이메일	연락처

연구책임자	박시현	중앙대학교 간호학과	조교수	sh8379@cau.ac.kr	010-4840-7483
공동연구자	고예정	공주대학교 간호학과 (이전: 광주대학교 간호학과)	부교수	486dpwjd@naver.com	010-8920-7556

6. 연구기간

본 연구의 예상 연구기간은 IRB 승인일로부터 2022년 2월 28일까지로 한다.

7. 연구대상자

본 연구의 대상자는 만 6세(취학 전 아동) 이하의 자녀를 양육하는 여성들로써 본 연구 참여에 대해 서면으로 동의한 자로 한다. 구체적인 선정기준은 다음과 같다.

1) 만 6세(취학 전 아동) 이하의 자녀를 양육하는 여성
2) 의학적 진단으로 정신분열병과 같은 중증정신질환 장애가 없으며 실험 시점에 항정신성 의약품을 복용하지 않는 자
3) 설문지의 내용을 이해하고 응답이 가능한 자
4) 화상 프로그램(Zoom)을 사용할 수 있는 자
5) 연구의 목적을 이해하고 참여를 서면으로 동의한 자

8. 예상 연구대상자 수와 산출 근거

연구 대상자 수는 G Power 3.1.2 program을 이용하여 산출한다. 선행연구인 강지숙(2017)의 웃음치료 메타 분석연구에서의 웃음치료의 효과크기는 정서적 영역 (0.912), 정신적 영역(0.735) 로 나타났다. 이에 본 연구에서는 효과크기(Effect size) 0.8, 유의수준(α) 0.05, 검정력(1-β) 0.80 으로 하여 적절한 대상자 수를 산출하며, 이에 필요한 최소인원은 각 집단(실험군 및 대조군) 당 26명으로 탈락자를 고려하여 총 70명을 모집할 것이다.

9. 연구대상자 모집

본 연구에서는 연구 참여자 모집 공고[부록 1]를 연구자 SNS 및 인터넷 맘카페 등에 업로드를 통하여 홍보를 진행하여 모집을 할 예정이다. 공고문을 본 대상자가 연구용 이메일[부록 2]로 연구에 대한 신청을 하면 연구자는 선정기준과 관련한 간단한 질의를 통해 연구 참여 대상자에 해당하는지 확인 한 후 본 연구의 대상자 조건에 맞을 시 연구 참여 설명문 및 동의서를 이메일로 발송할 것이다. 대상자는 설명문을 다 읽고 연구에 동의를 하여 동의서에 서명 후 연구자에게 송부하면, 실험 중재가 시작되는 일자와 시간 Zoom의 URL을 대상자의 휴대폰 문자로 발송한다. 이 때, 중재에 참여할 시 조용한 곳에서 자신의 마이크와 웹캠을 켠 상태에서 참여할 수 있도록 문자로 안내 할 예정이다.

10. 연구대상자 동의

예정된 실험 중재 진행 전, 연구자는 대상자들에게 연구 참여의 목적 및 17. 중지 및 탈락기준에 대하여 다시 구두로 설명할 예정이다.

11. 연구방법

1) 연구설계

본 연구는 영유아 자녀를 둔 어머니에게 적용한 비대면 방식을 활용한 온라인 웃음치료 프로그램이 어머니들의 불안, 우울 및 양육스트레스 감소에 미치는 효과를 검증하기 위한 무작위 대조군 전후 설계의 실험연구이다.

2) 연구 진행 절차 및 자료수집 방법

① 연구 승인절차

본 연구자는 연구진행에 앞서 연구윤리교육을 이수할 것이며 중앙대학교 IRB 승인을 받을 것이다.

② 연구자 준비 및 연구보조진행자 훈련

본 연구의 연구자 1인은 정신간호학 전공으로서 한국웃음임상치료센터에서 주관하는 웃음임상치료자 1급 자격을 소지하고 있다. 본 연구의 연구책임자는 연구설계 및 실험 전반을 검토 및 계획한다. 또한 웃음치료프로그램을 담당 할 강사 1인, 실험군과 대조군을 무작위 배정과 프로그램을 관리를 담당할 연구보조자 1인이 연구에 참여할 것이다. 또한 본 연구 진행 전 연구보조자에게 연구 진행과정 및 주의사항에 대해 교육을 실시할 것 이다.

③ 실험 진행 절차

중재 전 사전검사를 시행 한 후 실험군에게는 2주간의 개발된 Zoom을 활용한 웃음치료 프로그램을 적용하고 대조군에게는 Zoom을 통하여 연구자 및 웃음치료전문가가 선정한 오락프로그램을 시청하게 할 것이다. 중재 직후 실험군과 대조군에게 사후검사를 진행할 것이다. 본 연구자는 연구자 및 대상자에게 맹검법을 적용할 것이며 연구진행과정은 CONSORT 2010 가이드라인에 맞추어 진행할 것이다. 실험군과 대조군의 배정은 연구보조자 1인이 마이크로소프트 엑셀(Microsoft Excel)을 사용하여 단순 무작위 배정으로 실험군 35명 대조군 35명을 배정할 것이다. 실험군과 대조군에게는 할당은닉(Allocation concealment)를 하여 중재프로그램 시작 전 까지 배정 순서를 노출하지 않을 것이다.

본 연구는 Zoom을 활용하여 온라인 상에서 실험 중재프로그램을 진행할 예정이기 때문에 연구 참여자는 소음이나 방해가 없는 자신의 개인공간에서 참여할 것이다. 실험중재는 매주 화요일과 금요일에 주2회 2주간 진행하며 효과적인 중재를 위해 실험군은 자신의 마이크와 웹캠을 켜고 참여할 것이고 대조군의 경우 유머 프로그램 시청을 위해 마이크 음소거를 하고 진행할 것이다.

④ 사전조사

Google 온라인 설문을 통하여 일반적 특성, 불안, 우울 및 양육스트레스 정도를 측정할 것이다. 사전 조사 후에 실험군과 대조군으로 무작위 배정 할 것이다.

⑤ 실험처치: 비대면 방식의 웃음치료 프로그램 (Simulated laugher therapy)

실험군에게는 시뮬레이션 된 웃음(‘stimultated’ laugther)를 활용하여 연구자와 비대면 방식의 온라인 웃음 치료프로그램을 웃음치료학 석사학위를 취득하고 Zoom을 통해 웃음치료프로그램 운영 경험이 있는 전문강사와 함께 개발한 프로그램을 적용할 것이다. 프로그램은 온라인의 특성을 고려하여 대상자가 쉽게 따라 할 수 있는 웃음기법, 웃음율동을 활용하며 구성은 강지숙(2017)의 선행연구에서 4단계 구성프로그램(1.923)이 3단계 구성프로그램(0.720)보다 효과가 크고 웃음치료가 주요법(0.644)인 경우가 웃음을 보조적으로 병행한 요법(0.438)보다 효과크기가 큰 것으로 나타난 것을 근거로 하여 도입 10분, 전개 30분, 마무리 5분, 평가 5분의 4단계로서 웃음치료를 주 요법으로 하였다. 또한 웃음치료의 총 횟수, 회당 운영시간 총 시간이 길어질수록 웃음치료의 효과가 커지는 것이 아니며 주당 운영횟수가 많을수록 효과적이었다는 근거로 하여 1회 50분, 주 2회 2주간 적용한 총 4회로 구성되었다. 웃음 요법은 타인의 웃는 행위와 웃음소리를 통해 자연스러운 웃음을 유발할 수 있어 효과적이므로(이임선, 2012) 개인이 아닌 집단프로그램으로 개발하였다[부록 3]. 대조군에게는 맹검법을 적용하기 위해 실험군과 동일한 시간에 자발적 웃음(spontaneous laughter)을 유발할 수 있는 ‘코미디 빅

리그’ 라는 개그프로그램을 연구자가 선정한 후 줌을 통해 함께 시청하게 할 것이다.

⑥ 사후조사

2주간의 프로그램이 모두 종료 된 직후 실험군과 대조군에게 동일하게 Google 온라인 설문을 통하여 불안, 우울 및 양육스트레스 정도를 재 측정 할 것이다.

⑦ 질적조사 (Exit interview)

실험이 끝난 후 프로그램에 참여하였던 대상자 중 자발적인 지원자를 대상으로 프로그램 참여의 경험을 질적으로 조사한다. 이는 전화인터뷰 형식으로 진행될 예정이며 예상 인터뷰 소요시간은 30분~50분이다. 반구조적 질문지를 바탕으로 질문할 예정이며 질문지는 부록 4와 같다. 인터뷰는 대상자의 구두 동의하에 녹음되며 녹음된 인터뷰 내용은 모두 전사하여 분석에 이용될 예정이다.

12. 관찰 항목

Google 온라인 설문을 통하여 불안, 우울 및 양육스트레스의 변화를 실험 전후 관찰한다. 연구 도구는 다음과 같다[부록 5].

1) 연구도구

① 우울

어머니의 우울 정도 측정을 위하여 한국판 우울척도 CES-D(Center of Epidemiologic Studies Depression Scale)를 사용하여 측정하였다(전겸구 & 이민규, 1992). 총 21문항으로 구성되어 있으며, 각 문항은 4-likert 척도로 1=‘전혀 없었다’에서 4=‘매일 있었다’로 구성되어 있다. 점수가 높을수록 어머니의 우울 정도가 높음을 의미한다.

② 불안

어머니의 불안 정도 측정을 위하여 State-Trait Anxiety Inventory를 한덕웅, 이창소, 탁진국(1993)이 표준화한 척도를 사용하였다. 상태불안과 특성불안을 측정하는 문항이 각 20개로 총 40문항으로 구성되어 있고, 각 문항은 4-likert 척도로 1=‘전혀 그렇지 않다’에서 4=‘매우 그렇다’로 구성되어 있다. 점수가 높을수록 어머니의 불안 정도가 높음을 의미한다.

③ 양육스트레스

한국의 사회문화적 배경을 고려하여 김기현과 강희경(1997)이 개발한 양육스트레스 척도를 이용한다. 총 32문항으로 구성되어 5-likert로 체크하도록 구성되었다. 세부요인으로는 자녀양육으로 인한 일상적 스트레스(12문항), 부모역할수행에 대한 부담감(12문항), 타인양육으로 인한 죄책감(8문항)으로 구성되어 있다. 본 연구에서는 문항을 COVID-19 상황에 맞게 문항을 재구성 할 계획이며 자녀양육으로 인한 일상적 스트레스(12문항), 부모역할수행에 대한 부담감(12문항)만 측정한다.

13. 자료분석과 통계적 방법

양적 수집된 자료는 SPSS Win 25.0 을 이용하여 다음과 같이 분석할 예정이며 통계처리 후 가설검정에 대한 채택과 기각 여부는 유의수준 p= 0.05에서 검증할 것이다. 구체적인 방법은 다음과 같다.

1) 실험군과 대조군의 일반적 특성은 빈도와 백분율로 분석할 것이다.

2) 실험군과 대조군의 일반적 특성과 실험 전 종속변수에 대한 동질성 검증을 위하여 t-test를 사용하여 분석할 것이다.

3) 실험군과 대조군의 사전 - 사후의 변화량에 대한 두 집단의 차이 비교는 Shapiro-Wilk의 정규성 검정을 시행한 후 모든 변수가 정규성을 만족하면 Independent t-test를 사용하여 분석할 것이다.

질적 수집 자료는 Descriptive Content Analysis 방법을 통해 line-by-line으로 분석한다.

14. 예측 부작용 및 주의사항과 조치

웃음치료에 있어 신체적 및 정신적 부작용은 보고되지 않았다. 다만 본 연구의 특성 상 집에 고립되어 있는 대상자들을 만날 수 있기 때문에 혹여 연구에 참여한 대상자들 중 정신적인 문제나 가정 내 위험 요인(폭력 및 학대 등)에의 노출이 의심되는 경우 대상자와 따로 접촉할 수 있으며 대상자의 동의 후 필요한 조치(신고, 상담 및 지역사회 내 도움기관에 의뢰 등)를 할 수 있다.

15. 중지 및 탈락기준

본 연구의 참여 대상자는 자발적 의사에 따라 참여하고 연구 참여에 동의하지 않더라도 어떤 불이익도 받지 않는다는 사실과 연구 참여에 동의한 이후라도 자유의사에 의해서 원치 않을 시 참여를 중단하거나 동의를 철회할 수 있으며 그로 인한 불이익은 없을 것이라는 사실을 공지할 것이다.

또한 연구자의 사정에 의해서도 연구가 중지될 수 있고 연구 도중 지속적 연구 참여에 영향을 미치는 새로운 정보가 얻어지는 경우가 발생하였을 때는 수집된 내용과 개인 정보를 즉시 폐기할 것이라는 사실을 공지할 것이다. 연구 참여대상자는 중재에 4회 모두 참여하는 것을 원칙으로 하지만 자유의사에 따라 프로그램 중간에 참여를 원치 않는 경우에는 참여를 중단 할 수 있다.

16. 연구대상자의 이익 및 위험

웃음치료에 있어 대상자들이 겪을 수 있는 직접적인 위험은 보고된 바가 없다. 다만 앞서 기술한대로 연구에 참여한 대상자들 중 정신적인 문제나 가정 내 위험 요인(폭력 및 학대 등)에의 노출이 의심되는 경우 대상자와 따로 접촉할 수 있으며 필요한 조치(신고, 상담 및 지역사회 내 도움기관에 의뢰 등)를 할 수 있다. 본 프로그램을 완전히 이수한 대상자들에 감사의 표시로 20000원 상당의 참여금을 제공한다. 또한 Exit interview를 참여한 대상자에게는 10000원을 추가로 지급한다. 참여금은 연구자의 연구비에서 제공하므로 인건비 제공을 위한 정보를 대상자에게 요구할 수 있다. 그러나 이는 연구자가 따로 저장하지 않고 산학협력단에 그대로 제출 할 예정이며 이 때 취득하는 정보에 대해 미리 연구대상자에게 알려 동의하는 경우 연구에 참여하도록 한다.

17. 연구대상자 안전대책 및 개인정보보호대책

본 연구에서 취득된 자료는 연구의 목적으로만 사용함을 원칙으로 한다. 또한 본 연구는 기본적으로 모든 절차에서 익명으로 진행한다. 따라서 대상자 모집이나 자료 수집 시 대상자 식별정보(이름, 거주지, 직업, 소속 등)는 묻지 않을 것이며, 대상자의 정보는 임의의 ID를 제공하여 처리할 것이다. 수집된 연구자료는 비밀 잠금장치가 된 컴퓨터에 향후 3년 간 보관된 후 폐기될 것이다. Zoom url을 보내기 위해 대상자의 핸드폰 번호가 연구참여동의서를 받을 때 함께 취합될 예정이다. 그러나 대조군의 핸드폰번호는 실험 직후에, 실험군의 핸드폰 번호는 인터뷰 직후 바로 폐기할 예정이다.

18. 참고문헌

강지숙. (2017). 국내 학술지에 나타난 웃음치료 효과에 대한 메타분석. *예술인문사회융합멀티미디어논문지*, 7(1), 489-501.

김기현, 강희경. (1997). 양육스트레스 척도의 개발. 대한가정학회지 , 35(5), 141-150.

원지원, 강경선. (2018). 오르프 기법을 이용한 그룹음악치료가 워킹맘의 양육스트레스와 양육효능감에 미치

는 영향. 예술심리치료연구, 14(3), 115-135.

유현정, 배선혜, 우진승, 김경희, & 김광식. (2020). 원예치료가 코로나 19 로 인한 전업주부의 양육스트레스와 부모-자녀 상호작용에 미치는 효과. *인간식물환경학회 학술대회*, 248-248.

이임선, 정해성, 김경자, 정혜한, 권신영, 서은정, 정규철, 박애선, 오은영, 신수정, 은현주, 김향숙, 이선우 (2012). *웃음치료는 과학이다*. 다음생각.

전겸구, 이민규 (1992). 한국판 CES-D 개발연구 I. *한국심리학회지: 임상*,11(1), 65-75.

정혜인, 이근매. (2018). 집단미술치료 프로그램이 초등학생 어머니의 양육스트레스 감소에 미치는 효과. *임상미술심리연구*, 8(3), 27-45.

한덕웅, 이창호, 탁진국 (1993). Spielberger 상태-특성 불안 검사의 표준화. *학생지도연구*, 10(1), 214-222.

Derogatis, L.R. and Unger, R. (2010). Symptom Checklist‐90‐Revised. In The Corsini Encyclopedia of Psychology (eds I.B. Weiner and W.E. Craighead). https://doi.org/10.1002/9780470479216.corpsy0970

Foley, E., Matheis, R., & Schaefer, C. (2002). Effect of forced laughter on mood. *Psychological reports, 90*(1), 184-184.

Galea, S., Merchant, R. M.,& Lurie, N. (2020). The mental health consequences of COVID-19 and physical distancing: The need for prevention and early intervention. *JAMA Internal Medicine*, *180*(6), 817–818.https://doi.org/10.1001/jamainternmed.2020.1562

Kuru Alıcı, N., Zorba Bahceli, P., & Emiroğlu,O. N. (2018). The preliminary effects of laughter therapy on loneliness anddeath anxiety among older adults living in nursing homes: A nonrandomised pilot study. *International Journal of Older People Nursing*, *13*(4),e12206. https://doi.org/10.1111/opn.12206

Mora-Ripoll, R. (2011). Potential health benefits of simulated laughter: A narrative review of the literature and recommendations for future research. *Complementary Therapies in Medicine, 19*(3), 170-177.

Morishima, T., Miyashiro,I., Inoue, N., Kitasaka, M., Akazawa, T., Higeno, A., Idota, A., Sato, A.,Ohira, T., Sakon, M., & Matsuura, N. (2019). Effects of laughter therapy on quality of life in patients with cancer: An open-label, randomized controlled trial. *PLoS ONE*, *14*(6), 1–15.https://doi.org/10.1371/journal.pone.0219065

Ryu, K. H., Shin, H. S.,& Yang, E. Y. (2015). Effects of laughter therapy on immune responses inpostpartum women. *Journal of Alternative and Complementary Medicine*, *21*(12),781–788. https://doi.org/10.1089/acm.2015.0053

Torales, J., O'Higgins, M.,Castaldelli-Maia, J. M., & Ventriglio, A. (2020). The outbreak of COVID-19coronavirus and its impact on global mental health. *International Journal of Social Psychiatry*, *66*(4), 317–320.https://doi.org/10.1177/0020764020915212

Yim, J. E. (2016).Therapeutic benefits of laughter in mental health: A theoretical review. *Tohoku Journal of Experimental Medicine*, *239*(3), 243–249.https://doi.org/10.1620/TJEM.239.243

van der Wal, C. N., & Kok, R. N. (2019). Laughter-inducing therapies: Systematic review and meta-analysis. *Social Science & Medicine, 232*, 473-488.

연구 참여 설명문 및 동의서

<table>
<tr><td>연구제목</td><td colspan="5">COVID-19 시기 영유아기 자녀 어머니의 불안, 우울 및 양육스트레스 감소를 위한 비대면 웃음치료 프로그램의 적용 및 효과 검증</td></tr>
<tr><td>연구 기관/부서 및 주소</td><td colspan="5">• 연구기관: 중앙대학교 간호학과
• 주소: 서울시 동작구 흑석로 84 중앙대학교 간호학과</td></tr>
<tr><td rowspan="4">연구자</td><td rowspan="2">책임연구자</td><td>성명</td><td>박시현</td><td>소속</td><td>중앙대 간호학과</td></tr>
<tr><td>직위</td><td>조교수</td><td>전공분야</td><td>여성건강간호학</td></tr>
<tr><td rowspan="2">공동연구자</td><td>성명</td><td>고예정</td><td>소속</td><td>공주대 간호학과</td></tr>
<tr><td>직위</td><td>부교수</td><td>전공분야</td><td>정신간호학</td></tr>
</table>

연구 참여 권유

본 연구자는 귀하에게 **COVID-19 시기 영유아기 자녀 어머니의 불안, 우울 및 양육스트레스 감소를 위한 비대면 웃음치료 프로그램의 적용 및 효과 검증 연구**에 참여하시길 요청합니다. 귀하가 연구에 참여할 지, 않을 지 결정하는데 도움이 되도록 본 설명문은 연구의 목적, 내용, 위험 (불편감)이나 이익, 귀하의 정보를 포함한 자료관리 등에 대해 알려드리고자 합니다. 본 설명문을 주의 깊게 읽어보시고 궁금한 점이나 분명치 않은 점이 있으면 질문하여 주십시오.

1. 연구의 목적과 내용

2020년 초 경 발생된 코로나바이러스감염증-19 (이하 COVID-19)로 인하여 세계보건기구(WHO)가 감염증 최고 경보단계인 팬데믹을 선언하면서 여러 국가들이 감염예방 및 COVID-19의 영향을 최소화하고자 고군분투하고 있습니다. 이에, 여러 국가와 도시들은 바이러스의 감염전파 속도를 늦추고자 사람의 이동과 접촉을 제한하기 위해 사회적 거리두기 정책부터 도시 봉쇄(Shut down)까지 다양한 정책들을 펼치고 있습니다. 한국의 경우도 마찬가지로 정부가 주도하는 사회적 거리두기 정책을 펼치고 있으며 이로 인해 국민들은 ‘언택트(untact)’라는 새로운 시대를 경험하고 있습니다. 즉, 교육, 모임, 소비, 근무 등 대부분의 일상이 비대면 및 원격방식으로 전환되고 있으며 그로 인해 사람들 간의 대면 모임과 교류는 점차 줄어들고 있고 고립을 경험하는 인구집단이 늘어나고 있습니다.

이러한 시대적인 추세로 인해 최근 보건의 이슈는 감염병 예방, 역학 및 백신에 초점이 맞춰져왔으며 상대적으로 정신건강에 대한 부분이 간과되었던 것이 사실입니다. 그러나 최근 COVID-19 유행 초반 여러 정신건강 학자들이 경고한 바 대로 언택트로 인한 불안, 우울, 스트레스, 불면증, 분노, 공포 등 다양한 대중의 정신건강문제들이 수면 위로 떠오르고 있고 이는 ‘코로나 블루’라는 새로운 용어까지 만들어내게 되었습니다 (Galea, Merchant, & Lurie, 2020).

실제로 2003년 SARS-CoV나 2015년 MERS 유행 시에도 의료기관에서 일하는 의료진들을 중심으로 PTSD나 우울, 불안과 같은 정신과적 증상이 보고되었습니다(Torales et al., 2020). 또한 확진자를 접촉한 사람이나 감염자에 대한 대중들의 낙인과 회피 및 공포적 시선으로 인해 당사자들은 더욱 고통을 겪었다고 알려져 있습니다(Torales et al., 2020). COVID-19의 경우 앞선 두 바이러스 유행사례에 비해 월등히 높은 감염사례가 발생하고 있고 유행기간이 상당히 긴 점을 감안할 때 향후 정신건강위기의 대규모 발생이 예측되는 바입니다. 이에, COVID-19와 관련한 정신건강위기 예방 및 회복을 위한 비대면 방식의 중재가 시급하다고 하겠습니다.

특히 영유아를 양육하는 여성의 경우 정신건강의 위기가 높을 것으로 예상합니다. 본 연구자의 직전 연구에서 영유아를 양육하는 여성들은 COVID-19시대에 극심한 정신적 스트레스를 겪음을 보고하였습니다. 특히 이는 COVID-19가 가정의 경제적 문제, 육아도움기관이나 주변 도움인의 부재, 사회적 불안의 증가에 영향을 주고 이것이 여성들의 장시간의 양육, 주변으로부터의 전통적 아내/엄마 역할에 대한 기대 증가, 가정 내에서의 고립, 파트너와의 갈등 및 폭력, 사회적 지지의 감소 등으로 이어져 여성들의 정신건강 및 삶의 질에 부정적 영향을 초래하였습니다.

이에, 본 연구에서는 비대면 방식의 프로그램을 통해 영유아 자녀 어머니들의 불안, 우울 및 양육스트레스 감소에 미치는 효과를 검증하고자 합니다.

2. 연구 참여자의 연구 참여 절차 및 계획

1. 연구 참여 절차

연구참여에 의사를 보이고 이메일을 통해 연구자에게 연락한 대상자에게 <u>연구참여동의서 1부 및 인건비 지급신청서 1부</u>를 보내드립니다.

1) 연구참여동의서 작성 및 서명:

설명문을 다 읽고 연구에 그대로 동의를 하는 경우 동의서에 서명 후 연구자에게 송부하면, 실험 중재가 시작되는 일자와 시간 Zoom의 URL을 대상자의 휴대폰 문자로 발송해드립니다. 이 때, 프로그램 참여 시 조용한 곳에서 자신의 마이크와 웹캠을 켠 상태에서 참여할 수 있도록 문자로 안내 할 예정입니다.

2) 인건비 지급신청서 작성 및 서명:

본 연구의 4회기 프로그램에 모두 참여하는 경우 20000원의 인건비가 지급됩니다. 이는 산학협력단을 통해 지급되므로 인건비 지급 관련 서류작성이 요구됩니다. 이 때 성함, 주민번호, 주소, 계좌번호 등을 요구할 수 있습니다.

2. 연구계획

1) 사전조사 및 무작위 배정

프로그램 전후에 Google 온라인 설문을 통하여 참여 대상자들의 일반적 특성, 불안, 우울 및 양육스트레스 정도를 측정할 것 입니다. 사전 조사 후에 대상자들은 2개의 그룹으로 무작위 배정하여 진행 할 것입니다.

2) 사후조사

2주간의 프로그램이 모두 종료 된 직후 Google 온라인 설문을 통하여 불안, 우울 및 양육스트레스 정도를 재측정 할 것입니다.

3) 실험중재

본 연구는 Zoom을 활용하여 온라인상에서 실험 중재프로그램을 진행할 예정이기 때문에 연구 참여자는 소음이나 방해가 없는 자신의 개인공간에서 참여해야 합니다. 실험중재는 매주 화요일과 금요일 주2회 2주간 진행할 예정입니다.

4) 인터뷰 참여

실험이 끝난 후 프로그램에 참여하였던 대상자 중 자발적인 지원자를 대상으로 프로그램 참여의 경험을 질적으로 조사한다. 이는 전화인터뷰 형식으로 진행될 예정이며 예상 인터뷰 소요시간은 30분~50분이다. 반구조적 질문지를 바탕으로 질문할 예정이며 질문지는 부록 4와 같다. 인터뷰는 대상자의 구두 동의하에 녹음되며 녹음된 인터뷰 내용은 모두 전사하여 분석에 이용될 예정이다.

3. 본 연구와 관련된 위험 요소

웃음치료에 있어 대상자들이 겪을 수 있는 직접적인 위험은 보고된 바가 없습니다. 다만 연구 과정 중 참여자의 심각한 정신적 문제나 가정 내 위험 요인(폭력 및 학대 등)에의 노출이 의심되는 경우를 발견하면 대상자와 따로 접촉할 수 있으며 대상자의 동의 하에 필요한 조치(신고, 상담 및 지역사회 내 도움기관에 의뢰 등)를 할 수 있습니다.

4. 기밀 유지와 피험자 관련 자료에 대한 검토

본 연구에서 취득된 자료는 연구의 목적으로만 사용하며 외부에 배포하지 않는 것을 원칙으로 합니다. 또한 본 연구는 기본적으로 모든 절차에서 익명으로 진행합니다. 따라서 대상자 모집이나 자료 수집 시 대상자 식별정보(이름, 거주지, 직업, 소속 등)는 묻지 않을 것이며, 대상자의 정보는 임의의 ID를 제공하여 처리할 것입니다. 수집된 연구자료는 비밀 잠금장치가 된 컴퓨터에 향후 3년 간 보관된 후 폐기될 것입니다.

다만, 예외사항은 다음과 같습니다.

1) 연구의 수행과 자료의 신뢰성을 검증하기 위해 연구자 외에 연구 관련 모니터링 요원, 점검자, 기관위원회 및 정부 관련 부처장 등이 관련 규정이 정하는 범위 안에서 연구대상자의 비밀보장을 침해하지 않으며 연구대상자의 기록 등을 열람할 수 있습니다.

2) 연구 참여자들의 인건비 지급을 위해 필요한 정보를 대상자들에게 요구할 수 있습니다. 인건비 지급을 위해 필요한 정보는 이름, 주소, 주민번호, 계좌번호 등입니다. 그러나 이는 연구 자료에 포함되지 않으므로 연구자가 따로 저장하지 않고 산학협력단에 그대로 제출 할 예정입니다. 취득하는 정보에 대해 동의하는 경우 연구에 참여하도록 합니다.

3) 본 프로그램은 Zoom으로 진행되므로 Zoom url을 보내드릴 대상자의 핸드폰 번호를 묻고 있습니다. 그러나 이는 실험 및 인터뷰 종료 시 바로 폐기할 예정입니다.

5. 자의적 참여 및 연구 참여 동의를 철회할 권리

본 연구의 참여 대상자는 자발적 의사에 따라 참여함을 원칙으로 합니다. 연구 참여에 동의하지 않더라도 어떤 불이익도 받지 않을 것이며 연구 참여에 동의한 이후라도 자유의사에 의해서 원치 않을 시 참여를 중단하거나 동의를 철회할 수 있습니다. 그리고 그로 인한 불이익은 받지 않을 것입니다.

또한 연구자의 사정에 의해서도 연구가 중지될 수 있고 연구 도중 지속적 연구 참여에 영향을 미치는 새로운 정보가 얻어지는 경우가 발생하였을 때는 수집된 내용과 개인 정보를 즉시 폐기할 것입니다. 연구 참여대상자는 중재에 4회 모두 참여하는 것을 원칙으로 하지만 자유의사에 따라 프로그램 중간에 참여를 원치 않는 경우에는 참여를 중단 할 수 있습니다.

6. 기타 피험자의 인권 보호에 관하여 필요한 사항

본 4회기 프로그램을 완전히 이수한 대상자들에게는 20000원 상당의 참여인건비를 제공합니다. 참여금은 한국연구재단 연구비로 제공하므로 인건비 제공을 위한 정보를 대상자에게 요구할 수 있습니다.

본 연구에 대해 궁금하신 점이 있으시면 언제든지 아래의 연구자에게 연락 주십시오.

■ 연구책임자: 중앙대학교 간호대학 박 시 현 교수

Tel. 02-820-5737 (중앙대학교 박시현 교수 연구실)

본 연구에 참여하는 경우 귀하의 권리에 대한 질문이 있을 때에는 아래의 연락처를 이용하여 연구책임자에게 문의하십시오.

■ 중앙대학교 생명윤리 위원회 : TEL)02-820-6236, E-mail)jdhgo1@cau.ac.kr

연구 참여자 동의서

본인은 본 연구와 관련된 충분한 설명을 듣고 이해하였으며, 모든 궁금한 사항에 대하여 충분한 답변을 들었습니다. 충분한 시간을 갖고 생각한 이후에 본인은 상기 연구에 참여하기를 자발적인 의사에 의하여 동의합니다. 본인은 이메일을 통해 전자 동의서를 연구자에게 제출하며, 전자 동의서 및 이메일 송부 기록을 보관하여야 함을 이해하고 있습니다.

피험자 성명: ______________________(인) 날짜: ______.________.________

비대면 프로그램 참여를 위해 Zoom url을 받으실 핸드폰 번호: ________________________

연구자 성명: ______________________(인) 날짜: ______.________.________

책임연구자: 박 시 현 교수

전화번호: 02-820-5737

공동연구자: 고 예 정 교수

전화번호: 010-8920-7556

중앙대학교 생명윤리 위원회

TEL)02-820-6236, E-mail)jdhgo1@cau.ac.kr

[부록 1] 연구대상자모집 포스터

연구 참여자 모집 공고

COVID-19 시기 영유아기 자녀 어머니의 불안, 우울 및 양육스트레스 감소를 위한 비대면 웃음치료 프로그램의 적용 및 효과 검증

- **연구대상자 선정 기준:**

1) 만 6세 (초등학교 입학 전)의 자녀를 양육하고 있는 자
2) 연구결과에 영향을 줄 수 있는 중증정신질환 장애가 없으며 실험 시점에 향정신성 의약품을 복용하지 않는 자
3) 설문지의 내용을 이해하고 응답이 가능한 자
4) 화상 프로그램(zoom)을 사용할 수 있는 자
5) 연구의 목적을 이해하고 참여를 서면으로 동의한 자

- **실험 처치:** 비대면(zoom) 방식의 웃음치료 프로그램. 매주 화요일과 금요일에 주2회 2주간 진행 (총 4회기)

- **연구 참여 인건비:** 4회기를 완전히 이수한 대상자들에 한해 20,000원 지급

- **연구자:**

1) 중앙대학교 간호학과 박 시 현 교수
2) 광주대학교 간호학과 고 예 정 교수

- **신청 및 접수:**

1) 이메일 접수: dv.victim119@gmail.com

이메일에 연구 신청에 대한 의사를 주시면 연구참여동의서 및 연구 참여 인건비 지급을 위한 인건비 지급신청서를 보내드립니다. 동의서 서명 및 신청서 작성 후 연구자에게 보내주시면 접수 완료 됩니다.

- **문의:**

그 외 문의사항은 dv.victim119@gmail.com로 보내주세요.

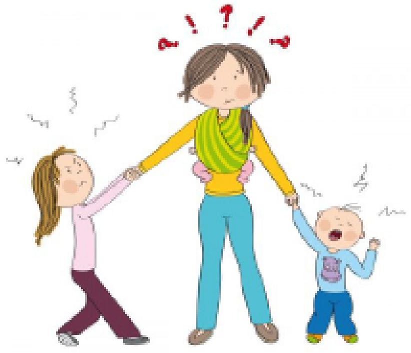

[부록 3] 대상자에게 연구참여설명문 및 동의서 발송 이메일 양식

안녕하십니까.

본 연구에 관심을 가져주셔서 감사드립니다.

저는 (중앙대학교 간호학과 박시현 교수) or (공주대학교 간호학과 고예정 교수)입니다. 본 연구는 연구책임자인 중앙대학교 간호학과 박시현 교수와 공동연구자인 공주대학교 간호학과 고예정 교수에 의해 수행되고 있습니다. 본 연구는 **COVID-19 시기 영유아기 자녀 어머니의 불안, 우울 및 양육스트레스 감소를 위한 비대면 웃음치료 프로그램의 적용 및 효과 검증 연구**입니다.

아래에 해당하시는 경우 연구 참여가 가능하니 이에 대한 부분을 읽어보시길 부탁드립니다.

1) 만 6세(취학 전 아동) 이하의 자녀를 양육하는 여성
2) 의학적 진단으로 정신분열병과 같은 중증정신질환 장애가 없으며 실험 시점에 항정신성 의약품을 복용하지 않는 자
3) 설문지의 내용을 이해하고 응답이 가능한 자
4) 화상프로그램(Zoom)을 사용할 수 있는 자
5) 화요일 및 금요일 오전에 프로그램 참여가 가능한 자
6) 연구의 목적을 이해하고 참여를 서면으로 동의한 자
7) 연구 참여금(20,000원) 인건비 지급을 위해 인건비 지급서류를 작성하여 제출하는 것에 동의하는 자

상기 항목들에 해당하신다면 연구와 관련한 설명문 및 동의서를 본 이메일에 첨부하오니 자세히 읽어보시기 바랍니다.

연구 참여에 그대로 동의하시는 경우 1) 연구참여설명문 맨 뒷장 동의서에 서명하시고, 2) 인건비 지급서류를 작성하시여 본 이메일로 회신주시면 연구 참여자로 등록이 됩니다. 프로그램 참여를 위한 zoom url은 이메일로 보내드리오니 늦지 않게 보내주시기 바랍니다.

[부록 3] 비대면 웃음치료 프로그램 컨텐츠 (4회기)

1 회차	주제	마음을 열고 웃기	
목표	웃음이 주는 효과를 알고 웃음 방법을 배운다.		
강의내용	단계	내용	시간
	도입<br>(인사 및 스트레칭)	-프로그램 소개<br>-자기소개 및 웃음 인사<br>-얼굴 표정 근육 스트레칭 및 입꼬리 올리기<br>-웃음 구호 외치기	10분
	전개<br>(웃음 운동)	웃음 기법<br>-성형외과 웃음<br>-8.4.2.1 안마 웃음<br>-사자 웃음<br>-박장대소와 파안대소 (노래 : 서울 구경)<br><br>웃음 율동 (노래 : 엄지 척)	30분
	마무리<br>(자기표현)	"나 대단한 사람이야"<br>자기 표현하기	5분
	평가<br>(감정 나누기)	감정 및 소감 나누기	5분

2 회차	주제	감사하기	
목표	자신의 인생을 긍정적으로 통합한다.		
강의내용	단계	내용	시간
	도입<br>(인사 및 스트레칭)	-자기소개 및 웃음 인사<br>-얼굴 표정 근육 스트레칭 및 입꼬리 올리기<br>-웃음 구호 외치기	10분
	전개<br>(웃음 운동)	웃음 기법<br>-거울 웃음<br>-치매예방 웃음<br>-핸드폰 웃음<br>-박장대소와 파안대소 (노래 : 서울 구경)<br><br>웃음 율동 (노래 : 따르릉)	30분
	마무리<br>(자기표현)	"~해서 감사합니다"<br>일상 속 긍정성 강화하기	5분
	평가<br>(감정 나누기)	감정 및 소감 나누기	5분

3 회차	주제	자존감 키우기	
목표	자아존중감 향상과 기분 전환이 된다.		
강의내용	단계	내용	시간
	도입<br>(인사 및 스트레칭)	-자기소개 및 웃음 인사<br>-얼굴 표정 근육 스트레칭 및 입꼬리 올리기<br>-웃음 구호 외치기	10분
	진행<br>(웃음 운동)	웃음 기법<br>-웃음 차 마시기<br>-웃음 총<br>-신문지 웃음<br>-박장대소와 파안대소 (노래 : 서울 구경)<br><br>웃음 율동 (노래 : 니가 왜 거기서 나와)	30분
	마무리	"나는 내가 정말 좋다"<br>자존감 키우기	5분
	평가<br>(감정 나누기)	감정 및 소감 나누기	5분

4 회차	주제	칭찬과 격려	
목표	대인관계 및 의사소통 능력이 향상 된다.		
강의내용	단계	내용	시간
	도입<br>(인사 및 스트레칭)	-자기소개 및 웃음 인사<br>-얼굴 표정 근육 스트레칭 및 입꼬리 올리기<br>-웃음 구호 외치기	10분
	전개<br>(웃음 운동)	웃음 기법<br>-건강 박수<br>-양치 웃음<br>-펜 테크닉 웃음<br>-박장대소와 파안대소 (노래 : 서울 구경)<br><br>웃음 율동 (노래 : 아모르파티)	30분
	마무리<br>(자기표현)	"나 그런 소리 많이 들어"<br>서로 간 칭찬과 격려 해주기	5분
	평가<br>(감정 나누기)	감정 및 소감 나누기	5분

[부록 4] zoom 기반 웃음치료 효과_ 질적분석을 위한 인터뷰 질문

1) 참여하시게 된 계기
2) COVID-19 시기 양육에서 힘든 부분은 무엇이었나요?
3) COVID-19 시기 양육과 관련한 정신적 스트레스 정도는 어떠하셨나요?
4) 4회기 프로그램에 참여하셨는데 참여하신 경험을 들려주세요.
5) 프로그램에서 어떤 점이 가장 좋으셨나요?
6) 느끼시기에 프로그램은 어떤 효과가 있던 것 같나요? 그렇게 느끼시는 이유는요?
7) 참여하시기 전과 후에 달라진 점이 있나요? (생활, 가족관계, 정신건강, 양육스트레스 등)
8) 달라진 이유는 무엇이라고 생각하나요?

[부록 5] 연구 설문지 (Google survey 이용 예정)

1. 우울 측정 설문지

다음은 일상생활에서 경험할 수 있는 내용들로 구성되어 있습니다. 각 문항을 잘 읽어보시고 지난 일주일동안 다음 내용이 얼마나 자주 일어났는지 해당하는 번호에 ○표 해 주십시오.

번호	문항	전혀 없었다	한두번 있었다	자주 있었다	매일 있었다
1	평소에는 아무렇지 않던 일이 귀찮게 느껴졌다.	1	2	3	4
2	입맛이 없었다. (별로 먹고 싶지 않다.)	1	2	3	4
3	가족이나 친구가 도와주어도 울적한 기분을 떨칠 수 없었다.	1	2	3	4
4	나는 다른 사람만큼 기분이 좋다.	1	2	3	4
5	하고 있는 일에 마음을 집중하기가 어려웠다.	1	2	3	4
6	기분이 우울했다.	1	2	3	4
7	하는 일마다 힘들게 느껴졌다.	1	2	3	4
8	미래에 대해 희망적으로 느꼈다.	1	2	3	4
9	내 인생은 실패라는 생각이 들었다.	1	2	3	4
10	두려움을 느꼈다.	1	2	3	4
11	잠을 설쳤다.	1	2	3	4
12	행복했다.	1	2	3	4
13	평소보다 대화를 적게 했다.	1	2	3	4
14	외로움을 느꼈다.	1	2	3	4
15	사람들이 나에게 다정하지 못하다고 느꼈다.	1	2	3	4
16	생활이 즐거웠다.	1	2	3	4
17	울었던 적이 있다.	1	2	3	4
18	슬픔을 느꼈다.	1	2	3	4
19	주위 사람들이 나를 싫어한다고 느꼈다.	1	2	3	4
20	무슨 일이든 제대로 할 수가 없었다.	1	2	3	4

2-3. 상태특성 불안 측정 설문지

다음 문항들은 당신이 지금 이 순간에 느끼고 있는 상태에 대한 내용들입니다. 각 문항을 잘 읽으신 후, 당신의 현재 느낌에 가장 일치하는 번호에 체크 해 주십시오.

번호	문항	전혀 없었다	한두번 있었다	자주 있었다	매일 있었다
1	나는 마음이 차분하다.	1	2	3	4
2	나는 마음이 든든하다.	1	2	3	4
3	나는 긴장되어 있다.	1	2	3	4
4	후회스럽고 서운하다.	1	2	3	4
5	나는 마음이 편하다.	1	2	3	4
6	나는 당황해서 어찌할 바를 모르겠다.	1	2	3	4
7	나는 앞으로 불행이 있을까봐 걱정하고 있다.	1	2	3	4
8	나는 마음이 놓인다.	1	2	3	4
9	나는 불안하다.	1	2	3	4
10	나는 편안하게 느낀다.	1	2	3	4
11	나는 자신감이 있다.	1	2	3	4
12	나는 짜증스럽다.	1	2	3	4
13	나는 마음이 조마조마하다.	1	2	3	4
14	나는 극도로 긴장되어 있다.	1	2	3	4
15	내 마음은 긴장이 풀려 포근하다.	1	2	3	4
16	나는 만족스럽다.	1	2	3	4
17	나는 걱정하고 있다.	1	2	3	4
18	나는 흥분되어 어쩔 줄 모르겠다.	1	2	3	4
19	나는 즐겁다.	1	2	3	4
20	나는 기분이 좋다.	1	2	3	4

다음 문항들 중에 당신이 일상생활에서 느끼는 일반적인 느낌과 가장 가까운 번호에 체크 해 주십시오.

번호	문항	전혀 없었다	한두번 있었다	자주 있었다	매일 있었다
1	나는 기분이 좋다.	1	2	3	4
2	나는 쉽게 피로해 진다.	1	2	3	4
3	나는 울고 싶은 심정이다.	1	2	3	4
4	나는 다른 사람처럼 행복했으면 한다.	1	2	3	4
5	나는 마음을 빨리 정하지 못해서 실패를 한다.	1	2	3	4
6	나는 마음이 놓인다.	1	2	3	4
7	나는 차분하고 침착하다.	1	2	3	4
8	나는 너무 많은 어려운 문제가 밀려 닥쳐서 극복할 수 없을 것 같다.	1	2	3	4
9	나는 하찮은 일에 너무 걱정을 많이 한다.	1	2	3	4
10	나는 행복하다.	1	2	3	4
11	나는 무슨 일이건 힘들게 생각한다.	1	2	3	4
12	나는 자신감이 부족하다.	1	2	3	4
13	나는 마음이 든든하다.	1	2	3	4
14	나는 위기나 어려움을 피하려고 애쓴다.	1	2	3	4
15	나는 울적하다.	1	2	3	4
16	나는 만족스럽다.	1	2	3	4
17	사소한 생각이 나를 괴롭힌다.	1	2	3	4
18	나는 실망을 지나치게 예민하게 받아들이기 때문에 머릿속에서 지워버릴 수가 없다.	1	2	3	4
19	나는 착실한 사람이다.	1	2	3	4
20	나는 요즘의 걱정거리나 관심거리를 생각만 하면 긴장되거나 어찌할 바를 모른다.	1	2	3	4

4. 양육스트레스 측정 설문지

다음은 아이를 키우면서 느끼는 어려움에 관한 내용입니다. COVID-19 가 발생한 이후 아이를 육아하는 부분과 관련하여 문항 별로 귀하의 평소 느낌이나 생각과 일치한다고 생각하시는 보기에 체크해주세요.

번호	문항	전혀 그렇지 않다	그렇지 않다	보통	그렇다	정말 그렇다
1	아이를 돌보는 것이 육체적으로 너무 힘들다.	1	2	3	4	5
2	아이를 돌보는 것이 너무 힘들어서 내 개인적인 시간을 가질 수 없다.	1	2	3	4	5
3	아이를 돌보느라 다른 집안일을 할 수가 없다.	1	2	3	4	5
4	아이 때문에 항상 마음에 여유가 없다.	1	2	3	4	5
5	시간에 맞춰 외출하기 위해 아이를 챙기는 일이 어렵다.	1	2	3	4	5
6	아이를 돌보기 위해 내 계획을 변경하는 경우가 많다.	1	2	3	4	5
7	아이와 함께 외출했을 때 아이를 다루기가 어렵다.	1	2	3	4	5
8	아이가 집을 너무 어지럽힌다.	1	2	3	4	5
9	아이를 매일 다른 사람에게 맡기고 출근하기가 어렵다.	1	2	3	4	5
10	아이가 태어난 이후로 잠자는 시간도 불규칙하다.	1	2	3	4	5
11	아이를 돌보기 위해 내 생활을 포기하는 경우가 많다.	1	2	3	4	5
12	아이가 태어난 이후 퇴근 후에 밀려 있는 집안일이 짜증스럽다.	1	2	3	4	5
13	좋은 부모가 될 수 있을지 확신이 서지 않는다.	1	2	3	4	5
14	아이를 잘 키울 수 있을지 자신이 없다.	1	2	3	4	5
15	내가 부모역할을 잘못하기 때문에 우리 아이가 다른 아이보다 뒤처지는 것 같은 기분이 들 때가 있다.	1	2	3	4	5
16	요즘은 육아정보가 너무 다양하여 어떤 것을 선택해야 할지 혼란스럽다.	1	2	3	4	5
17	아이를 더 친근하고 따뜻하게 대해야 한다는 것이 어렵게 여겨진다.	1	2	3	4	5
18	아이로부터 도망치고 싶을 때가 있다.	1	2	3	4	5
19	나를 성가시게 하는 일들이 몇 가지가 있다.	1	2	3	4	5
20	아이를 낳은 후 예전만큼 나의 생활이 즐겁지가 않다.	1	2	3	4	5
21	양육비용이나 탁아비용이 부담스럽다.	1	2	3	4	5
22	아이가 정서적으로 불안정한 모습을 보이면 내 탓인 것 같아 괴롭다.	1	2	3	4	5
23	아이 때문에 모임에 가면서도 그리 즐겁지 않을 것이라고 생각한다.	1	2	3	4	5
24	피곤할 때 아이가 놀아달라고 보채면 귀찮은 생각이 든다.	1	2	3	4	5

5. 일반적 특성 관련 질문

1) 귀하의 현재 결혼상태는 다음 중 어느 것에 해당합니까?
① 미혼
② 결혼 (초혼)
③ 결혼 (재혼)
④ 사별
⑤ 이혼
⑥ 별거

2) 귀하는 종교를 가지고 있으십니까?
① 가지고 있다.
② 가지고 있지 않다.

3) 귀하의 부부 관계와 관련하여 다음 문항 별로 가장 가까운 답에 체크해주세요.

문항	매우 불만족	비교적 불만족	보통	비교적 만족	매우 만족
1) 귀하는 배우자로서의 남편에 대하여 얼마나 만족하십니까?	1	2	3	4	5
2) 귀하는 결혼생활에 얼마나 만족하십니까?	1	2	3	4	5
3) 귀하는 남편과의 관계에 대해 얼마나 만족하십니까?	1	2	3	4	5
4) 귀하는 아버지로서의 남편에 대해 얼마나 만족하십니까?	1	2	3	4	5

4) 귀하의 남편이 양육에 참여하는 정도에 대해 다음 문항 별로 가장 가까운 답에 체크해주세요.

문항	전혀 그렇지 않다	별로 그렇지 않다	보통이다	대체로 그렇다	매우 그렇다
1) 남편은 아이에게 필요한 장남감이나 물품을 사다준다.	1	2	3	4	5
2) 남편은 아이의 습관이나 생활에 관심을 갖고 지도한다.	1	2	3	4	5
3) 남편은 아이에게 음식 또는 우유를 먹이거나 목욕을 시키는 등의 일을 한다.	1	2	3	4	5
4) 남편은 아이와 함께 자주 놀아주거나 이야기 상대가 되어 준다.	1	2	3	4	5

5) 귀하가 하루에 아이를 돌보는 데에 할애하는 시간을 총 몇 시간입니까?
주중: __________ 시간 ___________ 분
주말 혹은 공휴일: __________ 시간 ___________ 분

6) 귀하의 근무상태는 다음 중 어느 것에 해당합니까?
① 정근 (하루 8시간 이상 근무)
② 파트타임 근무 (하루 8시간 미만 근무)
③ 휴직 중

④ 전업주부

7) 귀하의 동거가족은 다음 중 어느 것에 해당합니까?
① 남편 + 본인 + 자녀
② 본인 + 자녀 (주말부부 혹은 이혼)
③ 시부모 + 남편 + 본인 + 자녀
④ 친정부모 + 남편 + 본인 + 자녀
⑤ 기타:

8) 귀하의 소득수준은 다음 중 어디에 가깝습니까?
① 상위
② 중상위
③ 중위
④ 중하위
⑤ 하위

9) 귀하의 아이의 양육형태는 어디에 가깝습니까?
① 엄마가 전적으로 24시간 양육
② 어린이집/유치원에서 하루 5시간 이상 양육 (25시간/주 이상)
③ 어린이집/유치원에서 하루 5시간 미만 양육 (25시간/주 미만)
④ 조부모로부터 하루 5시간 이상 양육 (25시간/주 이상)
⑤ 조부모로부터 하루 5시간 미만 양육 (25시간/주 미만)
⑥ 베이비시터로부터 하루 5시간 이상 양육 (25시간/주 이상)
⑦ 베이비시터로부터 하루 5시간 미만 양육 (25시간/주 미만)
⑧ 기타:

10) 귀하의 아이에게 스마트폰(혹은 테블릿 pc)을 보여주신 적이 있습니까?
① 한 번도 없다.
② 한 두 번 있다.
③ 일주일에 한 번 보여준다.
④ 일주일에 세 네 번 보여준다.
⑤ 거의 매일 보여준다.

11) 10번에 ②-⑤로 응답한 경우, 귀하의 아이가 스마트폰(혹은 테블릿 pc)을 한 번 시청할 시 몇 분 정도 시청합니까?
① 15분 이내
② 15분-30분 이내
③ 30분-1시간 이내
④ 1시간-3시간 이내
⑤ 아이가 스스로 그만 볼 때까지

12) 귀하의 나이는 몇 세입니까? ___________ 세

13) 자녀는 몇 명입니까? ______________ 명
14) 자녀의 나이는 각각 몇 세입니까? ______________세
15) 자녀의 성별은 무엇입니까?

첫째:
① 남
② 녀

둘째:
① 남
② 녀

끝.
