# Supplementary material for: A pilot randomized controlled trial of distance laughter therapy for mothers’ level of depression, anxiety, and parental stress during the COVID-19 pandemic
Source: PLoS One. 2023 Jul 14;18(7):e0288246. doi: 10.1371/journal.pone.0288246 (PMC10348554; doi:10.1371/journal.pone.0288246)
Supplement: S2 File — (PDF) [file pone.0288246.s003.pdf]

Application for Research Plan

(Translated version from Korean to English)

Research Title:

Application and Effectiveness of Non-face-to-face Laughter Therapy Program to Reduce the Anxiety, Depression, and Parenting Stress of Mothers Raising Infants and Toddlers During COVID-19

1. Background

The World Health Organization (WHO) declared a pandemic, the highest alert level for infectious diseases, due to COVID-19, which occurred around the beginning of 2020. As a result, many countries are struggling to prevent infection and minimize the impact of COVID-19. Consequently, numerous countries and cities have implemented diverse policies, including social distancing and shutdown, to limit people's movement and contact to slow the spread of the virus. The South Korean government is also implementing a social distancing policy, and as a result, South Koreans are experiencing a new era, called “untact”. Most people’s daily lives (e.g., education, gatherings, consumption, and work) are being converted to non-face-to-face and remote ways. Consequently, face-to-face meetings and exchanges between people are gradually decreasing, and a growing number of populations are experiencing isolation.

Due to this trend of the times, infectious disease prevention, epidemiology, and vaccines have been key health issues in recent years, whereas mental health has been relatively overlooked. However, as many mental health experts warned at the beginning of the COVID-19 pandemic, various mental health problems (e.g., anxiety, depression, stress, insomnia, anger, and fear) are emerging due to untact, and this created a new term, “corona-blue” (Galea et al., 2020).

In fact, psychiatric symptoms such as PTSD, depression, and anxiety were reported mainly in medical staff working in medical institutions during the 2003 SARS-CoV and 2015 MERS epidemics (Torales et al., 2020). It is also reported that those who contacted confirmed cases or those who were infected suffered from public stigma, avoidance, and fearful gaze (Torales et al., 2020).

Since COVID-19 has much more infection cases than the previous two virus epidemics and the duration of the COVID-19 pandemic is quite long, it is expected that there will be a large-scale outbreak of mental health crises. Thus, it is urgent to implement non-face-to-face interventions for preventing and recovering from mental health crises related to COVID-19.

It is anticipated that women raising young children are more vulnerable to mental health risks. The previous studies of the PIs reported that women raising infants reported increased mental distress in the era of COVID-19. Particularly, COVID-19 induced economic problems in many households, triggered the absence of childcare support agencies or helpers around them, and increased social anxiety. It also had a negative impact on women's mental health and quality of life because it caused lengthy childcare, increased expectations of the traditional wife/mother role from others, which led to isolation at home, conflict and violence with partners, and a decrease in social support.

The problems caused by the mother's high stress require social attention as it can impact on the child, and the family. The Office of Education is strengthening support for education-oriented services, such as parent education and parent training, as an intervention to mitigate parenting stress from several years ago. In addition, the Ministry of Gender Equality and Family is striving to create an environment that eases the burden of women's social life and childrearing by vitalizing step-by-step family education according to the life cycle. However, Jung & Lee (2018) revealed that there was a limit to reducing the burden of childrearing through educating and teaching parents (e.g., parenting skills), which emphasized the importance of psychological support for alleviating mothers' parenting stress. As a result, art therapy (Jung & Lee, 2018), music therapy (Won & Kang, 2018), and horticultural therapy (Yeu, Bae, Woo, Kim & Kim, 2020) have been proven as effective psychological interventions related to mothers' parenting stress. Therefore, it is necessary to expand research on various psychological interventions.

Laughter therapy is one of the cognitive behavioral therapies, and this intervention induces laughter using various mechanisms and draws positive emotions through it. Laughter can be divided into spontaneous laughter and simulated laughter (Mora-Ripoll, 2011). Spontaneous laughter refers

to laughter induced by humorous stimuli, such as jokes. It generally causes contraction around the orbit, and it is called a smile or humorous laughter. It is recognized as genuine laughter. Simulated humor is called fake laugh because it is self-triggered consciously (Foley, Matheis, & Schaefer 2002). Wal & Kok (2019) showed that interventions using simulated laughter were more effective than those using spontaneous laughter. Laughter therapy is in the form of a program that applies simulated laughter therapy. It has been applied to various subjects, including women with postpartum depression (Ryu, Shin, & Yang, 2015), cancer patients (Morishima et al., 2019), and older adults (Kuru Alıcı, ZorbaBahceli, & Emiroğlu, 2018). It has been shown to have a positive impact on diverse physical, mental, and social health outcomes, such as relieving muscle tension, reducing stress hormones, alleviating psychosocial symptoms, and improving self-esteem and quality of life (Yim, 2016). Laughter therapy is known to be highly applicable because it does not require special tools or places. However, only a few studies evaluated non-face-to-face laughter therapy. As untact-based mental health interventions are desperately needed in the present er, this study aims to examine the effects of a non-face-to-face laughter therapy program on the anxiety, depression, and parenting stress of women who are taking care of children for a long time due to COVID-19.

2. Objectives

The objective of this study is to examine the effects non-face-to-face laughter therapy on the anxiety, depression, and parenting stress of mothers with children during the social distancing period caused by COVID-19. The hypotheses of this study are as follows.

1) The treatment group, which has received non-face-to-face laughter therapy, will have less anxiety than the control group.
2) The treatment group, which has received non-face-to-face laughter therapy, will have less depression than the control group.
3) The treatment group, which has received non-face-to-face laughter therapy, will have less parenting stress than the control group.
Moreover, this study will identify the experience of participating in this program by using an exit interview.

3. Name and Address of Research Institute

Research Institute: Chung-Ang University

Address of Research Institute: Department of Nursing, Chung-Ang University, 84, Heukseok-ro, Dongjak-gu, Seoul, South Korea

4. Funding Agency

This study will be conducted with Kongju National University Research Grant.

5. Information of Principal Investigator, Co-Principal Investigator, and Contact Person

	Name	Affiliation	Position	Email	Tel
PI	Sihyun Park	Department of Nursing, Chung-Ang University	Assistant Professor	sh8379@cau.ac.kr	010-4840-7483
Co-PI	Yejung Ko	Department of Nursing, Kongju University	Associate Professor	486dpwjd@naver.com	010-8920-7556

6. Research Period

The period of this study will be from the IRB approval date to February 28, 2022.

7. Research Subjects

The subjects of this study shall be a woman who is raising a child ≤ 6 years (preschool child) and who gave written content to participate in the study. The specific selection criteria are as follows:

1) A woman who is raising a child $\leq$ 6 years (preschool child);
2) A person who had no psychiatric symptoms and medication histories at the time of intervention;
3) A person who can understand and respond to the items of the questionnaire;
4) A person who can use a video conference program (Zoom); and
5) A person who understands the objective of the study and has given written consent to participate.

8. Estimated Number of Subjects and Calculation Basis

The number of subjects is calculated using the G Power 3.1.2 program. Kang (2017) conducted a meta-analysis to evaluate laughter therapy and found that the effect size of laughter therapy was 0.912 for the emotional domain and 0.735 for the mental domain. Therefore, this study calculated the appropriate number of subjects based on effect size = 0.8, α = 0.05, and power (1-β) = 0.80. It is found that the minimum number of subjects required for each group (the treatment group and the control group) is 26 people, and 70 subjects will be recruited considering the dropouts.

9. Recruitment of Subjects

This study will recruit study participants by uploading the participant recruitment announcement to the researchers' SNS and Internet Mom Cafe. If a person reads the announcement and applies for the study through the email, the research will confirm whether the person is eligible for the through a series of simple questions related to the selection criteria. Then, if the person meets the selection criteria of this study, the informed consent procedure will be performed through the email. If the person agrees to participate in the study, the person have to sign the consent form, scan, and send it to the researcher. Then, the date and time when the experimental intervention starts and the URL of Zoom will be sent to the person's mobile phone as a text message. It will be guided by a text message that when the person (subject) will participate in the intervention, the person shall participate in a quiet place while turning on the microphone and webcam.

10. Subjects' Consent

Before conducting the scheduled experimental intervention, the researcher will verbally explain to the subjects the objective of the study and 17. Suspension and Dropout Criteria.

11. Materials and Methods

1) Experimental design

This pilot is a randomized controlled pre-test and post-test trial to evaluate the effects of an online laughter therapy program on the anxiety, depression, and parenting stress of mothers with infants or toddlers.

2) Study procedure and data collection methods

① Study approval procedure

The researchers will complete the research ethic education courses prior to conducting the study and will receive IRB approval for this study from Chung-Ang University.

② Researcher preparation and training of research assistants

One researcher majored in psychiatric nursing and has a first-class clinical laughter therapist certificate issued by the Korea Laughter Clinic Academy. The PI of this study will review and plan the experimental design and overall experiment. Moreover, one instructor who will be in charge of the laughter therapy program and one research assistant who will be in charge of randomly assigning subjects to the treatment group and the control group and managing the program will participate in the study. In addition, the research assistant will be educated on the research procedure and precautions before conducting this study.

③ Experiment procedure

After conducting a pre-test before the intervention, the treatment group will receive the developed laughter therapy program using Zoom for 2 weeks, while the control group will watch an entertainment program selected by researchers and laughter therapy experts through Zoom. Immediately after the intervention, a post-test will be conducted for the treatment group and the control group. The researcher will apply the blinding method to the researchers and subjects, and the study will be conducted according to the CONSORT 2010 guidelines. One research assistant will randomly assign the subjects to the treatment group (35 subjects) and control group (35 subjects) by using Microsoft Excel. Allocation concealment will be applied to the treatment group and the control group so that the allocation order will not be disclosed until the intervention program starts.

Since this study will carry out an experimental intervention program online using Zoom, the participants will participate in this study in their own personal space without noise or disturbance. The treatment mediation will be conducted twice a week on Tuesdays and Fridays for two weeks. For effective intervention, the treatment group will participate with their microphones and webcams turned on, and the control group will mute their microphones while watching entertainment programs.

④ Pre-test (preliminary survey)

This study will measure general characteristics, anxiety, depression, and levels of parenting stress through Google online questionnaire. The subjects will be randomly assigned to the treatment group and the control group after the preliminary survey.

⑤ Experimental treatment: Non-face-to-face simulated laugher therapy

The treatment group will receive the non-face-to-face online laughter therapy program, which was developed by the researcher and a professional instructor who earned a master's degree in laughter therapy and has experience in operating a laughter therapy program through Zoom, by using “simulated laughter”. The program is composed of four stages (10 minutes for introduction, 30 minutes for implement, 5 minutes for wrap-up, and 5 minutes for evaluation). According to Kang (2017), the 4 stage program (1.923) was more effective than the 3 stage program (0.720). The effect size was larger when laughter therapy was the main method (0.644) than when laughter therapy was used supplementarily (0.438). The program utilizes laughter techniques and laughter

rhythms that the subject can easily follow considering that this program is an online program. Moreover, it consists of 4 sessions (50 minutes each), applied twice a week for two weeks. Laughter therapy is effective because it can induce natural laughter through the laughing action and voice of others (Lee, 2012), so it was developed as a group program rather than an individual program. To apply the blinding method to the control group, the researcher will select episodes of “Comedy Big League”, a comedy program, which can induce spontaneous laughter, and make the subject watch them through Zoom while the treatment group is receiving the treatment.

⑥ Post-test

Immediately after the end of the two-week program, the levels of anxiety, depression, and parenting stress of the treatment group and the control group will be measured again by using the same Google online questionnaire.

⑦ Qualitative test (Exit interview)

After the experiment is over, a qualitative test will be conducted on the experience of participating in the program using volunteers among the participants who participated in the program. This will be conducted in the form of a telephone interview, and the estimated duration of the interview is 30 to 50 minutes. The questions will be based on the semi-structured questionnaire. Interviews will be recorded upon the verbal consent of the subjects, and all recorded interviews will be transcribed and used for analysis.

12. Observation Items

This study will observe the changes in anxiety, depression, and parenting stress before and after the experiment by using Google online surveys. The research tools are as follows:

1) Research tools

① Depression

The degree of the mother's depression will be measured using the Korean version of the CES-D (Center of Epidemiologic Studies Depression Scale) (Jeon & Lee, 1992). It consists of 21 items, and each item is measured using a 4-Likert scale (1 = “not at all” to 4 = “every day”). A higher score means a higher degree of the mother's depression.

② Anxiety

The degree of the mother's anxiety will be measured by the standardized State-Trait Anxiety Inventory Scale (Han, Lee, and Tak, 1993). It consists of 40 items (20 items measuring state anxiety and 20 items measuring trait anxiety), and each item is measured using a 4-Likert scale (1 = "not at all" to 4 = "every day"). A higher score means a higher degree of mother's anxiety.

③ Parenting stress

The parenting stress scale developed by Kim and Kang (1997) will be used by considering the socio-cultural background of Korea. It consists of 32 items and was configured to measure it using a 5-Likert scale. The detailed factors include daily stress due to raising children (12 items), the burden of parental role (12 items), and guilt due to raising children by others (8 items). This study will restructure the items to suit the COVID-19 situation, and measure only the daily stress due to parenting (12 items) and the burden of performing parental roles (12 items).

13. Data Analysis and Statistical Methods

The quantitatively collected data will be analyzed as follows using SPSS Win 25.0, and the significance of the hypotheses will be determined at p = 0.05. The specific methods are as follows:

1) The general characteristics of the treatment and control groups will be analyzed by descriptive statistics.
2) Independence t-test will be used to compare the changes of depression, state/trait anxiety, and parenting stress after applying the intervention between the two groups.
3) Shapiro-Wilk's normality test will be performed to test the normality of variables.

The collected qualitative data will be analyzed line-by-line by using the descriptive content analysis method.

14. Anticipated Side Effects, Precautions, and Countermeasures

No physical or psychological side effects due to laughter therapy are reported. In case we find a subject suspected to have a mental health crisis or be exposed to a risk factor (e.g., violence and abuse) at home during the study, the subject may be contacted separately. With the subject's consent, necessary interventions (e.g., report, consultation, and request to a relevant agency in the community) can be taken.

15. Suspension and Dropout Criteria

The subjects of this study will participate in this study voluntarily. The subjects participating in this study will be informed that they will not receive any disadvantages even if they do not agree to participate in the study. They can suspend participation or withdraw their consent if they do not want it of their free will even after agreeing to participate in the study without any resulting disadvantages.

They will be also informed that the study may be suspended due to the circumstances of the researcher. The collected data and personal information will be immediately destroyed if new information influencing continued research participation is found during the study period. In principle, the subjects shall participate in all four interventions, but if they do not wish to participate in the study in the middle of the program of their free will, they may discontinue participation.

16. Benefits and Risks of Subjects

No direct risk on subjects due to laughter therapy has been reported. However, as described above, if it is suspected that any of the subjects participating in the study have a mental problem or are exposed to a risk factor (e.g., violence and abuse) at home, the subject may be contacted separately and necessary intervention (e.g., report, counseling, and request to a relevant agency in the community) can be taken.

As an incentive, KRW 20,000 will be provided to those who have completed this program. Moreover, an additional KRW 10,000 will be paid to those who participate in the exit interview. Researchers will request to the subject to provide information needed for the incentive payment. However, it will be submitted to the institution with being concealed, and the researcher will not

save it personally. The information obtained from the subjects will be notified in advance, and the subjects will participate in the study only when they agree to provide the information.

17. Safety Measures and Privacy Protection for Subjects

The data obtained in this study will be used only for research purposes. Moreover, all procedures of this study will be conducted anonymously. Therefore, when recruiting subjects or collecting data, subject identification information (e.g., name, residence, occupation, and income) will not be asked, and subject information will be processed by using a random ID. The collected research data will be stored in a computer with a secret lock for three years and then destroyed. The cell phone number of the subject will be collected when the subject submits the research consent form to send the Zoom URL. The cell phone number of the control group will be discarded immediately after the experiment, and the cell phone number of the treatment group will be discarded immediately after the interview.

18. References

강지숙. (2017). 국내 학술지에 나타난 웃음치료 효과에 대한 메타분석. *예술인문사회융합멀티미디어논문지*, 7(1), 489-501.

김기현, 강희경. (1997). 양육스트레스 척도의 개발. *대한가정학회지*, 35(5), 141-150.

원지원, 강경선. (2018). 오르프 기법을 이용한 그룹음악치료가 워킹맘의 양육스트레스와 양육효능감에 미치는 영향. *예술심리치료연구*, 14(3), 115-135.

유현정, 배선혜, 우진승, 김경희, & 김광식. (2020). 원예치료가 코로나 19 로 인한 전업주부의 양육스트레스와 부모-자녀 상호작용에 미치는 효과. *인간식물환경학회 학술대회*, 248-248.

이임선, 정해성, 김경자, 정혜한, 권신영, 서은정, 정규철, 박애선, 오은영, 신수정, 은현주, 김향숙, 이선우 (2012). *웃음치료는 과학이다*. 다음생각.

전겸구, 이민규 (1992). 한국판 CES-D 개발연구 I. *한국심리학회지: 임상*,11(1), 65-75.

정혜인, 이근매. (2018). 집단미술치료 프로그램이 초등학생 어머니의 양육스트레스 감소에 미치는 효과. *임상미술심리연구*, 8(3), 27-45.

한덕웅, 이창호, 탁진국 (1993). Spielberger 상태-특성 불안 검사의 표준화. *학생지도연구*, 10(1), 214-222.

Derogatis, L.R. and Unger, R. (2010). Symptom Checklist–90–Revised. In The Corsini Encyclopedia of Psychology (eds I.B. Weiner and W.E.

Craighead). https://doi.org/10.1002/9780470479216.corpsy0970

Foley, E., Matheis, R., & Schaefer, C. (2002). Effect of forced laughter on mood. *Psychological Reports, 90*(1), 184-184.

Galea, S., Merchant, R. M., & Lurie, N. (2020). The mental health consequences of COVID-19 and physical distancing: The need for prevention and early intervention. *JAMA Internal Medicine*, *180*(6), 817-818.https://doi.org/10.1001/jamainternmed.2020.1562

Kuru Alıcı, N., Zorba Bahceli, P., & Emiroğlu,O. N. (2018). The preliminary effects of laughter therapy on loneliness anddeath anxiety among older adults living in nursing homes: A nonrandomised pilot study. *International Journal of Older People Nursing*, *13*(4), e12206. https://doi.org/10.1111/opn.12206

Mora-Ripoll, R. (2011). Potential health benefits of simulated laughter: A narrative review of the literature and recommendations for future research. *Complementary Therapies in Medicine, 19*(3), 170-177.

Morishima, T., Miyashiro,I., Inoue, N., Kitasaka, M., Akazawa, T., Higeno, A., Idota, A., Sato, A.,Ohira, T., Sakon, M., & Matsuura, N. (2019). Effects of laughter therapy on quality of life in patients with cancer: An open-label, randomized controlled trial. *PLoS ONE*, *14*(6), 1-15.https://doi.org/10.1371/journal.pone.0219065

Ryu, K. H., Shin, H. S., & Yang, E. Y. (2015). Effects of laughter therapy on immune responses inpostpartum women. *Journal of Alternative and Complementary Medicine*, *21*(12),781-788. https://doi.org/10.1089/acm.2015.0053

Torales, J., O'Higgins, M., Castaldelli-Maia, J. M., & Ventriglio, A. (2020). The outbreak of COVID-19coronavirus and its impact on global mental health. *International Journal of Social Psychiatry*, *66*(4), 317-320.https://doi.org/10.1177/0020764020915212

Yim, J. E. (2016). Therapeutic benefits of laughter in mental health: A theoretical review. *Tohoku Journal of Experimental Medicine*, *239*(3), 243-249.https://doi.org/10.1620/TJEM.239.243

van der Wal, C. N., & Kok, R. N. (2019). Laughter-inducing therapies: Systematic review and meta-analysis. *Social Science & Medicine, 232*, 473-488.
